# A High Precision Survey of the Molecular Dynamics of Mammalian Clathrin-Mediated Endocytosis

**DOI:** 10.1371/journal.pbio.1000604

**Published:** 2011-03-22

**Authors:** Marcus J. Taylor, David Perrais, Christien J. Merrifield

**Affiliations:** 1 Medical Research Council Laboratory of Molecular Biology, Cambridge, United Kingdom; 2 Université de Bordeaux, Interdisciplinary Institute for Neuroscience, UMR 5297, Bordeaux, France; 3 CNRS, Interdisciplinary Institute for Neuroscience, UMR 5297, Bordeaux, France; The Scripps Research Institute, United States of America

## Abstract

Dual colour total internal reflection fluorescence microscopy is a powerful tool for decoding the molecular dynamics of clathrin-mediated endocytosis (CME). Typically, the recruitment of a fluorescent protein–tagged endocytic protein was referenced to the disappearance of spot-like clathrin-coated structure (CCS), but the precision of spot-like CCS disappearance as a marker for canonical CME remained unknown. Here we have used an imaging assay based on total internal reflection fluorescence microscopy to detect scission events with a resolution of ∼2 s. We found that scission events engulfed comparable amounts of transferrin receptor cargo at CCSs of different sizes and CCS did not always disappear following scission. We measured the recruitment dynamics of 34 types of endocytic protein to scission events: Abp1, ACK1, amphiphysin1, APPL1, Arp3, BIN1, CALM, CIP4, clathrin light chain (Clc), cofilin, coronin1B, cortactin, dynamin1/2, endophilin2, Eps15, Eps8, epsin2, FBP17, FCHo1/2, GAK, Hip1R, lifeAct, mu2 subunit of the AP2 complex, myosin1E, myosin6, NECAP, N-WASP, OCRL1, Rab5, SNX9, synaptojanin2β1, and syndapin2. For each protein we aligned ∼1,000 recruitment profiles to their respective scission events and constructed characteristic “recruitment signatures” that were grouped, as for yeast, to reveal the modular organization of mammalian CME. A detailed analysis revealed the unanticipated recruitment dynamics of SNX9, FBP17, and CIP4 and showed that the same set of proteins was recruited, in the same order, to scission events at CCSs of different sizes and lifetimes. Collectively these data reveal the fine-grained temporal structure of CME and suggest a simplified canonical model of mammalian CME in which the same core mechanism of CME, involving actin, operates at CCSs of diverse sizes and lifetimes.

## Abstract 2

The molecular dynamics of clathrin-mediated endocytosis in living cells has been mapped with an approximately ten-fold improvement in temporal accuracy, yielding new insights into the molecular mechanism.

## Author Summary

The molecular machinery of clathrin-mediated endocytosis concentrates receptors at the cell surface in a patch of membrane that curves into a vesicle, pinches off, and internalizes membrane cargo and a tiny volume of extracellular fluid. We know that dozens of proteins are involved in this process, but precisely when and where they act remains poorly understood. Here we used a fluorescence imaging assay to detect the moment of scission in living cells and used this as a reference point from which to measure the characteristic recruitment signatures of 34 fluorescently tagged endocytic proteins. Pair-wise comparison of these recruitment signatures allowed us to identify seven modules of proteins that were recruited with similar kinetics. For the most part the recruitment signatures were consistent with what was previously known about the proteins' structure and their binding affinities; however, the recruitment signatures for some components (such as some BAR and F-BAR domain proteins) could not have been predicted from existing structural or biochemical data. This study provides a paradigm for mapping molecular dynamics in living cells and provides new insights into the mechanism of clathrin-mediated endocytosis.

## Introduction

Clathrin-mediated endocytosis (CME) is the principal means by which mammalian cells internalize cell surface receptors (reviewed in [1]). Some 40 years of electron microscopy (EM), genetic, and biochemical studies are distilled in the canonical model of CME [2] (reviewed in Figure S1). Here, interaction of receptors with adaptor proteins stabilise nascent clathrin-coated pits (CCPs) at random sites on the plasma membrane [3]. Growing CCPs acquire cargo and invaginate via clathrin polymerization [4] and the coordinated action of curvature-inducing/sensing BAR [5] and F-BAR domain proteins [6],[7], ENTH domain proteins [8], and possibly actin [9]–[11]. The neck of the deeply invaginated CCP is severed in a mechanism involving the large GTPase dynamin [12],[13], and possibly a phosphoinositide (PI) phosphatase [14], to release a clathrin-coated vesicle (CCV), which uncoats through the action of GAK/auxilin [15],[16].

Understanding how the multiple components of CME are spatially and temporally organized is a challenging problem that has been tackled using live-cell fluorescence microscopy (reviewed in [2],[17]). In a typical experiment using dual colour total internal reflection fluorescence microscopy (TIR-FM), the recruitment dynamics of fluorescent protein (FP)–tagged endocytic proteins were measured relative to the disappearance of spot-like CCPs, which was used as a fiducial marker to indicate internalization [6],[18],[19]. Using this strategy the recruitment dynamics of endocytic proteins were coarsely grouped into “early” and “late” relative to CCP disappearance [20] (Figure S1), but finer temporal resolution was not possible because the moment of scission, the endpoint of the invagination process, was unknown. In addition to spot-like CCPs, larger clathrin patches were also observed at the substrate proximal surface of many cell types, where they were variously thought to participate in the canonical pathway of CME [4],[21] or cell adhesion [22],[23], or were thought to represent endocytic intermediates in an actin-dependent mode of endocytosis distinct from the canonical pathway of CME [23].

To circumvent the subjective classification of endocytically active clathrin-coated structures (CCSs), a TIR-FM assay was invented to detect single scission events directly by monitoring the accessibility of pH-sensitive fluorescent CCP cargo to rhythmically imposed changes in extracellular pH (the “pulsed pH” [ppH] assay [10], reviewed in Figure S2). Surprisingly, it was discovered that scission events were hosted by spot-like CCPs, as predicted from the canonical model, and also by larger clathrin patches (collectively referred to as CCSs [10]), thus raising questions about what characterises endocytically active CCS at optical resolution.

The following study was designed to explore the fine-grained temporal structure of late stages of the mammalian CME machinery using TIR-FM and the ppH assay. First, scission events were mapped to their host CCSs to determine what dynamic characteristics defined endocytically active CCSs. It was found that CCSs of diverse size and lifetimes hosted scission events that engulfed comparable amounts of receptor cargo, and CCSs could either disappear (“terminal events”) or persist (“non-terminal events”) following scission. Second, we assessed the accuracy of CCS disappearance as a fiducial marker for internalization and showed it introduced an error comparable to the time course of CCS invagination and CCV formation. It was thus necessary to use the ppH assay to obtain a precise measurement of recruitment dynamics. Third, we surveyed the recruitment dynamics of a representative set of 34 mammalian endocytic proteins to sites of scission and derived, for each protein, a characteristic “recruitment signature” by aligning and averaging ∼1,000 recruitment traces per protein. A cluster analysis of recruitment signatures revealed the modular organization of the CME machinery, similar to yeast [24], while closer inspection revealed unanticipated features of some signatures. Finally, scaling relationships between CCS size and lifetime and the cohort of endocytic proteins recruited to scission events were explored. It was found that the same set of proteins was recruited in the same order to scission events at diverse dynamic classes of CCSs, although subtle scaling relationships between CCS size and protein recruitment were identified.

Collectively these data provide, to our knowledge, the highest resolution temporal map of the late stages of mammalian CME to date. This map (1) suggests a simplified model of mammalian CME in which the same core mechanism can operate at both spot-like CCSs and larger clathrin patches observed with fluorescence microscopy, (2) illustrates the similar modular organization of mammalian and yeast endocytosis, and (3) proves that recruitment dynamics of endocytic proteins such as the F-BAR protein FBP17 and BAR domain protein SNX9 cannot always be predicted from biochemical or structural properties.

## Results

### Detection of Scission Events at CCSs

**Figure 1:**
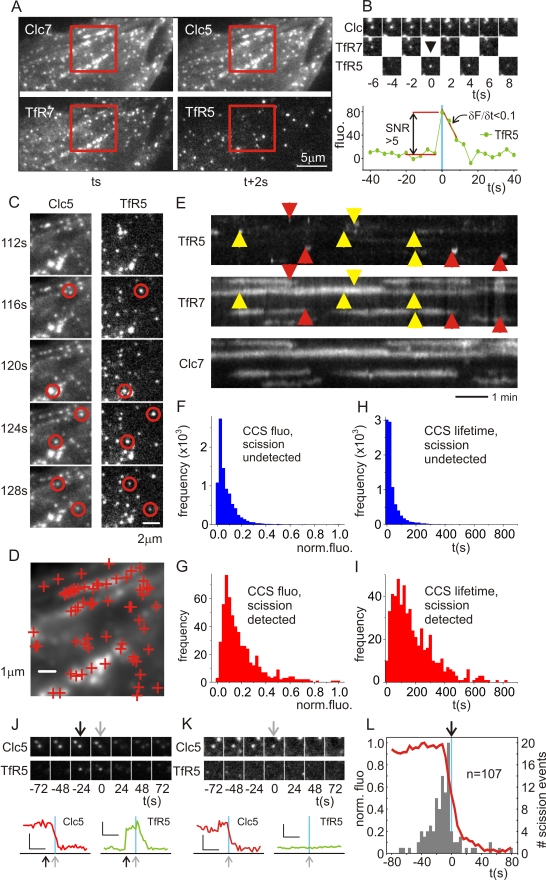
The characteristics of endocytically active CCSs. (A) Images from a sequence of 400 images acquired in synchrony with alternating pH. Portion of an NIH-3T3 cell expressing Clc-mCherry (top panels) and TfR-phl (lower panels) imaged at pH 7.4 (Clc7, TfR7; left panels) at time *t* and at pH 5.5 (Clc5, TfR5; right panels) at time *t*+2 s. (B) Example scission event. A CCS (Clc spot, upper panel) colocalized with a patch of TfR-phl at pH 7.4 (TfR7, middle panel). The scission event manifested as the appearance of a spot of pH-insulated TfR-phl in images acquired at pH 5.5 (TfR5, middle panel). Bona fide scission events met thresholds for SNR (SNR >5) and post-scission slope (Δ*F*/Δ*t *<0.1; see Materials and Methods for details). (C) Time-resolved images of region of interest in (A). Scission events (red circles) manifested as the appearance of pH-insulated TfR-phl spots at both punctate CCSs and larger, pleiomorphic CCSs. (D) Spatial map of scission events in region of interest from (A). Candidate scission events (red crosses) detected over a 10-min interval were plotted on the average Clc-mCherry image. Scission events tended to cluster at “hot spots”. (E) A kymograph of Clc7 and TfR7 objects graphically illustrates that Clc7 and TfR7 at CCSs co-varied over time. Scission events appeared as transient streaks in the TfR5 image series (arrowheads). Two types of scission events occurred: those associated with complete disappearance of the associated CCS (terminal events, red arrowheads) and those where the CCS persisted (non-terminal events, yellow arrowheads). (F–I) To explore the characteristics of scission-competent CCSs further, the segmented Clc7 objects were tracked and divided into scission-detected” and scission-undetected CCSs (see Materials and Methods). (F and G) Histograms of median normalised fluorescence of Clc-mCherry for scission-undetected CCSs (F) and scission-detected CCSs (G). (H and I) Histograms of lifetimes for scission-undetected CCSs (H) and scission-detected CCSs (I). (J–L) Comparison of CCS disappearance and scission events as fiducial markers for CME. (J) A CCS disappearance event (grey arrow) with an associated scission event (black arrow). (K) An example CCP disappearance (grey arrow) without an associated scission event. Of 197 disappearance events, 107 (54%) were associated with scission events, as predicted. (L) CCS disappearance (red line) versus the timing of scission (grey histogram) for 107 scission-detected CCS disappearance events (timing of scission relative to CCS disappearance: −7±22 s).

To detect CME scission events at CCSs, NIH-3T3 cells were transiently transfected with Clc-mCherry and TfR-phl and assayed using the ppH assay, as described previously [10]. A large-diameter perfusion tip was brought close to the target cell, and perfusate was cycled between buffer of pH 7.4 and pH 5.5 in synchrony with image acquisition at 0.5 Hz (see [10] and Figure S2). In an image acquired at arbitrary time point *t*, at pH 7.4, TfR-phl concentrated in spots and patches of Clc-mCherry and free in the plasma membrane fluoresced brightly (Figure 1A). When the perfusate was switched to pH 5.5 and an image was acquired 2 s later (at *t*+2 s), TfR-phl fluorescence at the plasma membrane was quenched and revealed bright punctae of pH-insulated TfR-phl sequestered in internal vesicles, while Clc-mCherry fluorescence remained unchanged (Figure 1A). The cycle of pH switching and image acquisition was repeated to generate an image series acquired at alternating high and low pH. Scission events manifested as the abrupt appearance of TfR-phl spots in images acquired at pH 5.5, colocalized with Clc-mCherry-labelled CCSs (Figure 1B; Video S1). Although it took 4 s to complete a cycle of pH change, the precision with which scission events were detected was ∼2 s because, for an event to be detected, scission had to occur in a ∼2-s time window at pH 7.4 prior to detection at pH 5.5 (see [10] and Figure S2). We could therefore align the red fluorescence traces, acquired at 0.5 Hz, with an accuracy of 2 s. Visual inspection revealed that scission events were associated with both punctate CCSs and also larger, pleiomorphic clathrin patches (Figure 1C; Video S1), and events could occur repeatedly at larger CCSs, as shown previously [10] (Figure 1D). Larger CCSs may represent flat clathrin lattices, with peripheral invaginations, or clusters of smaller CCSs too close to resolve by optical microscopy [25],[26]. Inspection of kymographs revealed that Clc-mCherry and TfR-phl patches waxed and waned in synchrony at both small and large CCSs, demonstrating the similarity of these two signals and suggesting that TfR7 fluorescence could be used as a surrogate signal to report the relative size or lifetime of CCSs (Figure 1E). Scission events were not always associated with the disappearance of the host CCS, and, similar to previous findings, events were either terminal (where the spot-like CCS disappeared following scission, red arrows in Figure 1E) or non-terminal (where CCS persisted following scission, yellow arrows in Figure 1E) [10].

To analyse large numbers of scission events we developed a semi-automated analysis pipeline to identify candidate events, screen for bona fide events, and quantify the fluorescence changes associated with these events in both the green and red channels. The purpose of this screening strategy was not to detect all scission events in an image series but to impose stringent selection criteria and automatically sample a large proportion of genuine scission events. The criteria for selection of bona fide scission events included persistence of the TfR5 spot, association with a “host” CCS, adequate signal-to-noise ratio (SNR), and slope of the TfR5 signal following appearance (Figure 1B, see Materials and Methods for details).

### The Dynamic Characteristics of Endocytically Active CCSs

To quantitatively investigate the characteristics of endocytically active CCSs we detected scission events in seven cells expressing Clc-mCherry and TfR-phl and identified a set of 851 bona fide events. First we analysed the relationship between the relative amount of TfR-phl localized at a CCS (TfR7 fluorescence), the relative amount of clathrin (Clc fluorescence), and the relative amount of TfR-phl internalized by a scission event (TfR5 fluorescence). As expected, there was a significant correlation between TfR7 fluorescence and Clc7 fluorescence (Spearman's rho = 0.85, *p*<0.05; Figure S3), showing that larger CCSs contained more TfR-phl cargo, and indicating that CCS size could be estimated using TfR7 fluorescence. However, there was no significant correlation between Clc7 and TfR5 fluorescence (Spearman's rho = −0.0024, p>0.05) or between TfR7 and TfR5 fluorescence (Spearman's rho = −0.0022, *p*>0.05; 3). Therefore, and consistent with both visual inspection of the current data and previous results [10], the amount of cargo internalized by scission events was independent of the size of the host CCS, and endocytically active CCSs could be either spot-like structures or larger, pleiomorphic clathrin patches. In mechanistic terms, this is consistent with the relatively constant dimensions of coated invaginations viewed by EM whether they occurred in isolation, as part of a cluster, or as a peripheral invagination at a flat patch of clathrin [25],[26]. To check that extracellular acidification did not affect the size of clathrin-coated invaginations, we fixed cells under control conditions and after exposure to acidic buffer for 1 min or 10 min, and imaged them using thin section EM (Figure S3). Under both control and acidified conditions the clathrin-coated invaginations were of relatively uniform size, with a maximum dimension of ∼100 nm (Figure S3F–S3I).

Next we explored what dynamic characteristics defined endocytically active CCSs. All CCSs present in the Clc-mCherry dataset (seven cells) were tracked using a multi-particle tracking algorithm, similar to previous studies [27] (see Materials and Methods), yielding a set of 11,447 track histories. For each CCS track history the fluorescence of Clc-mCherry was quantified, and the CCS track histories were classified according to the presence or absence of scission events, wherein a track history was defined as scission detected if a bona fide scission event fell within five pixels, or 500 nm. The median normalised Clc-mCherry fluorescence of scission detected CCSs was significantly greater than for scission undetected CCSs (0.190 versus 0.078, *p*<0.05; Figure 1F and 1G), and the median lifetime of scission-detected CCSs was longer than the lifetime of scission-undetected CCSs (189 s versus 38 s, *p*<0.05; Figure 1H and 1I). Therefore, scission events defined a class of larger, longer-lived CCSs. The shorter-lived scission-undetected CCSs most likely correspond to the “abortive” CCSs described previously [3],[27],[28], although some of these structures may have represented endosomal clathrin.

For NIH-3T3 fibroblasts the average time between de novo appearance of a spot-like CCS and the first detected scission event was previously found to be ∼100 s [10]. This was similar to previous estimates in BSC1 cells, wherein productive CCSs were defined as spot-like CCSs having lifetimes anywhere from tens to hundreds of seconds (average 87 s) [3],[27],[28]. Because the size and lifetimes of scission-detected CCSs were so variable (Figure 1H and 1I), in our subsequent investigation of late events in CME we made measurements over a time window of ±80 s, centred on scission.

### The Recruitment Signatures of 34 Endocytic Proteins to Sites of Scission with a Temporal Resolution of 2 s

In previous analysis of the molecular dynamics of CME, the disappearance of spot-like CCSs was used as a fiducial marker to indicate endocytic events [6],[18],[19]. However, we discovered that CCS disappearance gave an inaccurate and imprecise estimate of scission, with a temporal uncertainty comparable to the time course of CCS invagination and CCV formation [10] (−7±22 s; *n* = 107; six cells) (Figure 1J and 1K). CCS disappearance most likely corresponded to CCV uncoating and/or movement, and if CCS disappearance was used as a fiducial marker for CME the waveform of aligned and averaged recruitment signatures would be significantly smeared. We hypothesized that measuring the recruitment of endocytic proteins with improved temporal accuracy might reveal otherwise hidden temporal structure in the CME mechanism, and so we measured the recruitment signatures of a representative set of 34 mammalian endocytic proteins relative to scission.

First, and to illustrate the experimental strategy and details of the analysis, we determined the kinetics of dynamin1 recruitment relative to scission. The Dyn1-mCherry signals acquired at pH 5.5 and pH 7.4 were corrected for bleed through and interlaced, and confidence intervals were calculated on the fluorescence recruitment signature using a randomization procedure (–S4D). As a negative control for protein recruitment we assayed caveolin1-mCherry, which forms spot-like structures at the plasma membrane but which is not enriched at sites of CME [29] (Figure S4E–S4H).

**Figure 2:**
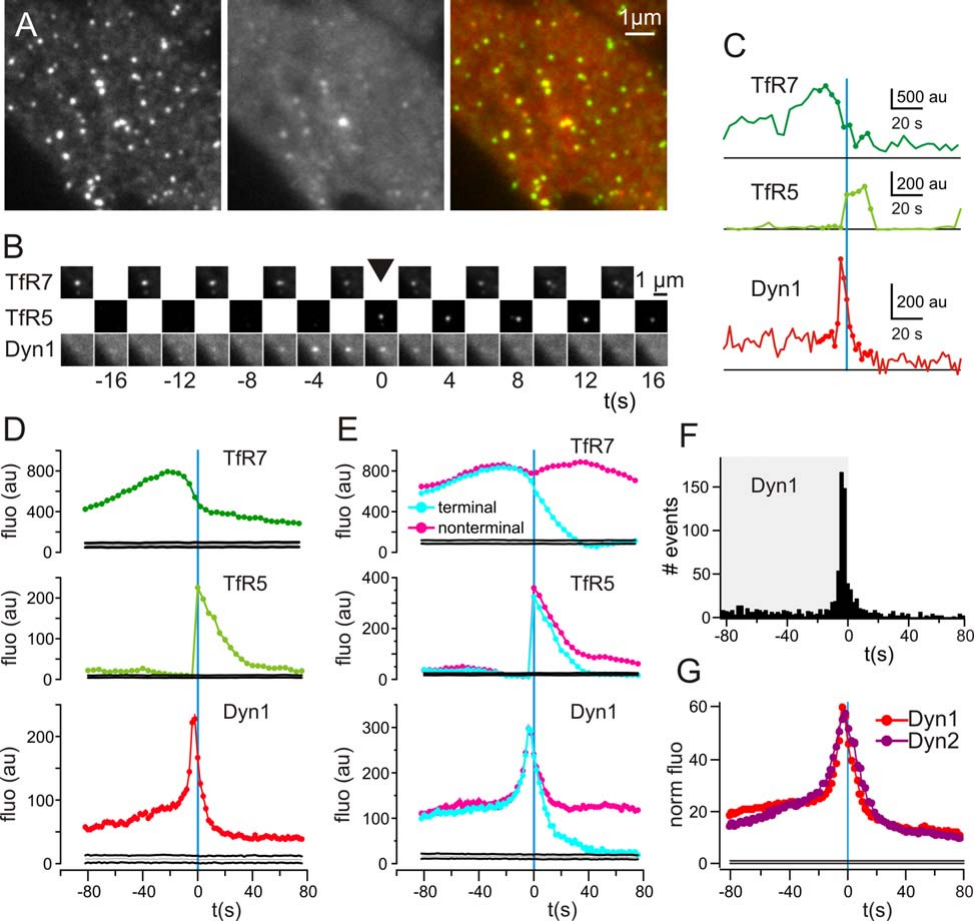
Dynamin was recruited at the time of CCV formation. (A) Portion of a NIH-3T3 cell co-transfected with TfR-phl (left) and Dyn1-mCherry (Dyn1, centre), observed with TIR-FM at pH 7.4. Dynamin1 was colocalized with a subset of TfR-phl patches (yellow dots in the merged image, right). (B) An example scission event. At time 0, a CCV was detected in the image at pH 5 (black arrowhead). Dynamin1 was recruited transiently, with a peak at time −4 s. (C) Fluorescence measurements (dark green, TfR7; light green, TfR5; red, dynamin1) corresponding to the event displayed in (B). The dots correspond to the images shown. Vertical blue line shows time = 0 and horizontal lines show fluorescence = 0. (D) Average fluorescence for TfR7, TfR5, and dynamin1 for the events detected in the cell shown in (A) (*n* = 290). Black lines represent the median and 95% confidence limits for random fluorescence measurements (see Materials and Methods for calculation). (E) Data as in (C) pooled for eight cells (1,297 events). Averages of fluorescent traces of terminal (light blue) and non-terminal events (magenta) for TfR7, TfR5, and dynamin1. Note the overlap of curves before time = 0. (F) Histogram of peak dynamin1 recruitment for individual events. (G) Average dynamin1 (red, eight cells) and dynamin2 (purple, six cells) fluorescence curves normalised to the randomized measures. au, arbitrary units.

Dynamin is essential for scission [30], and it is thought to be recruited in the last steps of vesicle formation [18],[19]. Cells co-transfected with TfR-phl and Dyn1-mCherry and imaged with TIR-FM microscopy at pH 7.4 showed punctuate patterns that were partially colocalized (Figure 2A), and scission events, localized to patches of TfR-phl marking CCSs (Figure 2B), were frequently (75%) preceded by a transient burst of Dyn1-mCherry (Figure 2B and 2C). Examination of the average fluorescence traces revealed that the TfR7 signal dropped before scission, which might indicate progressive polarization of receptor cargo in the invaginating CCS similar to AP2 [31] (Figure 2D). The average recruitment signatures of Dyn1-mCherry showed a peak 2 to 4 s before vesicle detection (Figure 2D–2F), which corresponded to the time of vesicle creation. Before this transient burst Dyn1-mCherry was, on average, present at low levels on the CCS, as seen in the average and in individual examples, consistent with previous observations [32] (Figure 2B and 2D). Visual inspection revealed that pre-scission recruitment of Dyn1-mCherry manifested as low-amplitude “flickering”, which persisted following scission in non-terminal events, consistent with continued recruitment of Dyn1-mCherry to the remaining portion of CCSs at the plasma membrane (Figure 2E). Strikingly, the temporal spread of Dyn1-mCherry average fluorescence (∼8 s) and peak recruitment around scission (Figure 2D) was much narrower than when CCS disappearance was used as a reference for CCV creation (∼20 s) [18],[19]. Finally, the recruitment kinetics of Dyn2-mCherry was very similar to that of Dyn1-mCherry (Figure 2G).

**Figure 3:**
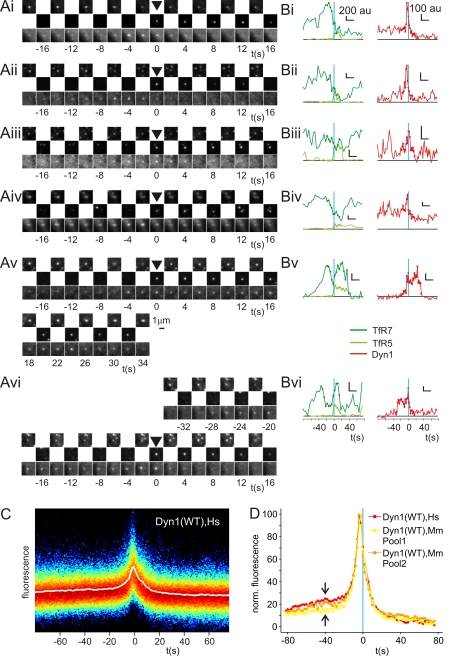
Individual Dyn1-mCherry recruitment events were variable, but the average Dyn1-mCherry recruitment signature was stable. (A and B) Natural variation of Dyn1-mCherry recruitment to sites of scission. (Ai–Avi) Consecutive images of TfR7 (top), TfR5 (middle), and Dyn1-mCherry (bottom) movies centred on scission events detected in the TfR5 movies. The data are from the cell shown in Figure 2A. (Bi–Bvi) Quantification of fluorescence for TfR5 (light green curves), TfR7 (dark green curves), and Dyn1 (red curves) images for the corresponding events shown in (A). Vertical blue lines indicate *t* = 0 s, and black horizontal lines indicate zero fluorescence. Horizontal scale bar corresponds to 20 s, fluorescence values as indicated. Dots correspond to frames shown in (A). (C) The full set of Dyn1-mCherry fluorescence traces were normalised and overlaid as a cloud plot (see Materials and Methods). Red indicates higher data density, blue, lower density, and black, background. The average fluorescence recruitment trace is indicated by a white line. (D) Replicate Dyn1-mCherry recruitment signatures for either human (Hs) or mouse (Mm) Dyn1-mCherry. au, arbitrary units; WT, wild type.

Visual inspection revealed heterogeneity among individual Dyn1-mCherry fluorescence traces (Figure 3A and 3B). To explore whether there was any evidence for natural sub-classes of recruitment signature, the full set of Dyn1-mCherry recruitment traces was normalised and overlaid to generate a cloud plot (Figure 3C). The average fluorescence recruitment trace followed the highest data density, and there was no obvious evidence of bifurcations or the presence of “natural” sub-classes of Dyn1-mCherry recruitment traces (Figure 3C). Therefore, the heterogeneity apparent among individual traces was largely unstructured and most likely represented natural noise rather than mechanistic differences between scission events.

To further test the reproducibility of the Dyn1-mCherry average recruitment signature two datasets were generated using either human or mouse Dyn1-mCherry. For human Dyn1-mCherry seven cells were analysed (1,276 events), and for mouse Dyn1-mCherry 21 cells were analysed, arbitrarily divided into two pools of 10 cells (Pool 1, 2,126 events) and 11 cells (Pool 2, 2,622 events). The average recruitment signatures for human Dyn1-mCherry-transfected cells and either pool of mouse Dyn1-mCherry-transfected cells were very similar (correlation coefficient >0.95), with only minor differences in the pre-scission offset (Figure 3D). Therefore, although individual Dyn1-mCherry fluorescence recruitment traces were variable, the average Dyn1-mCherry recruitment signatures were reproducible and remarkably stable.

Next, we applied the ppH protocol and analysis to an additional set of 33 mammalian endocytic proteins fused to mCherry (Figure S5). To generate an overview of the molecular dynamics of CME we chose a range of proteins that included well-established players (e.g., dynamin and GAK), proteins with tentative or poorly understood links to CME (e.g., Eps8 [33]), and proteins with established links to endocytosis in yeast and which we hypothesized should be recruited to sites of endocytosis in mammalian cells (e.g., cofilin and coronin [34],[35]). Of the 34 endocytic proteins analysed, only the recruitment signature of cortactin had been previously measured with a temporal resolution of 2 s, and the recruitment dynamics of the other 33 proteins remained uncharacterised at this resolution. A reverse transcription PCR (RT-PCR) analysis revealed that all proteins except ACK1, amphiphysin1, CIP4, and FCHo1 were expressed in fibroblasts (Figure S6). It remains possible that the expression of such a diverse set of endocytic proteins is peculiar to cultured cells and would not normally be seen in native tissue. For example, dynamin1 is thought to be expressed predominantly in neurons [36], although low levels of dynamin1 expression have been detected in primary mouse fibroblasts, and the expression level in fibroblast cell lines was found to increase upon immortalization [9]. However, and as described previously [15],[23],[27],[37],[38], we expected that proteins expressed in fibroblasts and heterologously expressed proteins would still incorporate into the CME machinery and could thus reveal useful information.

For each protein we generated red FP (RFP) fusion constructs and assayed 5–7 cells per construct using the ppH protocol, yielding a dataset of ∼1,000 bona fide scission events per protein type (Table S1). Overexpression of mCherry-tagged proteins may perturb the recruitment dynamics of endocytic proteins or have other deleterious effects on the endocytic machinery. Therefore, to ameliorate the possible effects of overexpression cells were transiently co-transfected with TfR-phl and the relevant RFP chimera ∼48 h prior to the experiment, and cells with only the lowest 10%–20% levels of expression used for imaging experiments. In our experience this procedure gave the most consistent results, and target cells showed no overt changes in morphology. Although the incidence rate of scission events varied up to 5-fold between constructs (Table S1), variability between cells expressing the same construct was also high and cells expressing low levels of a selection of RFP fusion proteins still internalized Tfn-A647 (Figure S7). Moreover, and by definition, the ppH assay measured the dynamics of protein recruitment only to successful scission events.

**Figure 4:**
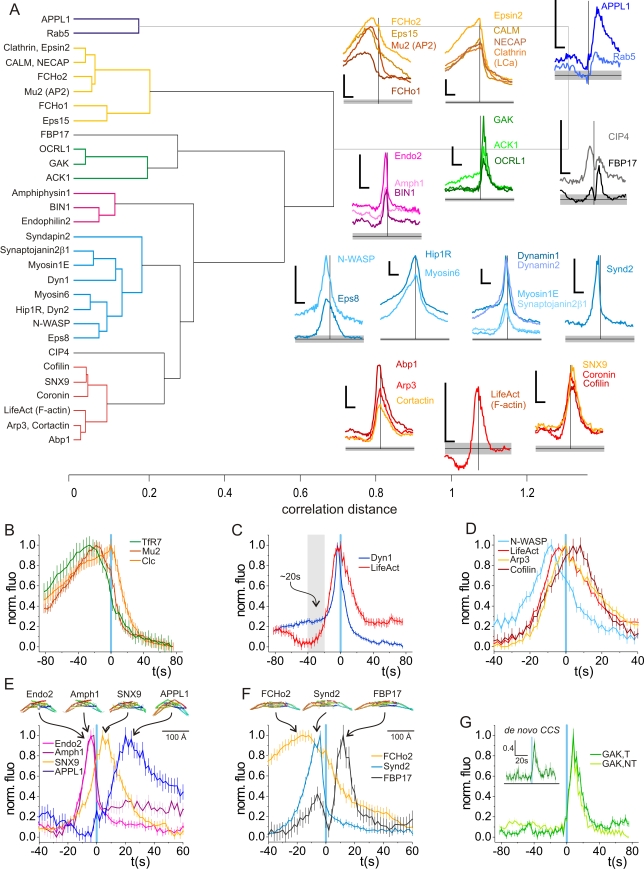
Modules of endocytic proteins. (A) Left, a list of proteins with significant recruitment to CCVs used in this study. The proteins were clustered according to the correlation distance between recruitment signatures shown at right. Clusters with distance below 0.2 are marked with a colour defining a protein module. The two Toca proteins, CIP4 and FBP17, fell outside of any cluster but were grouped together according to biological arguments (see text). Right, average recruitment signatures normalised to their randomized measures. Vertical black line shows time = 0. Horizontal black lines show median randomized measures, taken as fluorescence origin, and grey areas the 95% upper and lower confidence intervals. Scale bars = 20 s (horizontal) and 10×95% confidence interval (vertical). (B–F) Detailed view of recruitment traces of (B) TfR7, mu2 (AP2), and Clc; (C) Dyn1 and lifeAct; (D) N-WASP, lifeAct, Arp3, and cofilin; (E) endophilin, amphiphysin, SNX9, and APPL1; and (F) FCHo2, syndapin2, and FBP17. (G) GAK for terminal events (T), non-terminal events (NT) and scission events at spot-like CCSs that formed de novo (inset).

The recruitment signatures of each protein were assessed, and the full set of traces compared pair-wise and organized in a dendrogram by hierarchical clustering (Figure 4; the full set of fluorescence recruitment signatures is shown in Figure S8 and peaks histograms in Figure S9). This analysis revealed, similar to previous results in yeast [24], that natural groups or clusters were formed based on the similarity of recruitment signatures. In each of the seven groups or modules there were proteins expected to show similar recruitment signatures on the basis of previous knowledge (i.e., previous imaging studies, known binding affinities, and known biochemical properties), while some patterns of recruitment were unexpected. A brief comparison of key predictions, based on a priori models, and actual observations follows below.

### Clathrin, Adaptor Proteins, and Receptor Cargo

The clathrin recruitment signature, reported by Clc-mCherry, showed a slow build up that peaked at scission and dropped sharply thereafter, presumably as the newly formed vesicle uncoated (Figure 4A and 4B; although note the different signatures of terminal and non-terminal events, Figure S8). Most similar to clathrin were the PI(4,5)P2-binding epsin N-terminal homology domain (ENTH)/AP180 N-terminal homology domain (ANTH) adaptor proteins epsin and CALM [8],[39], both of which directly bind clathrin. Surprisingly, NECAP, which has a high affinity for the AP2α-ear [40], displayed a similar recruitment profile to clathrin rather than AP2.

Other adaptor proteins formed a distinct subgroup within the clathrin/adaptor protein module. Based on previous work it was predicted that AP2 fluorescence (marked by mu2-mCherry) should markedly decrease before scission, indicating the polarized segregation of AP2 in the nascent bud [31] and/or loss from developing buds before clathrin [41] (though see [42]). This was indeed the case, and, in addition, the adaptor protein Eps15, TfR7 (i.e., the receptor cargo), and the F-BAR domain proteins FCHo1 and FCHo2 showed similar signatures, suggesting that these proteins were also polarized and/or lost from the developing bud before clathrin (Figure 4A, 4B,and 4F). This latter observation may be consistent with a recently proposed role for FCHo proteins in CCP nucleation and the generation of curvature early in bud formation [43].

### The Dynamin/Myosin Module

Dynamin was present at low levels on CCSs at all times, and a burst of recruitment preceded scission (Figures 2, 4A, and 4C). Other proteins showed a similar pattern of biphasic recruitment and thus defined a dynamin module. These included actin-binding proteins such as the actin- and clathrin-binding protein Hip1R [44] as well as the motor protein myosin6, which binds actin and the adaptor protein Dab2 [45]. Other proteins involved in actin dynamics and grouped in the dynamin module included the Arp2/3 activator N-WASP [46],[47], Eps8, an actin capping protein that forms a complex with Abi1 and binds N-WASP [33] and the motor protein myosin1E [48]. The PI(4,5)P2 phosphatase synaptojanin2β1, which binds to the NBAR domain protein amphiphysin1 [49], had recruitment kinetics similar to those of dynamin and peaked at scission but showed little recruitment at time points before −20 s (Figure 4A). Finally, the F-BAR protein syndapin2 [50],[51], which binds dynamin and N-WASP, was recruited early, peaking at −4 s before being quickly discarded following scission (Figure 4A and 4E). The rapid loss of syndapin2 signal may be due to collapse of the highly curved membrane neck at the moment of scission.

The improved temporal accuracy of the ppH assay allowed us to re-evaluate the temporal relationships between dynamin recruitment and actin dynamics. Earlier work suggested that dynamin and actin were recruited sequentially to sites of scission [18]. Here, a more accurate comparison of dynamin and actin recruitment revealed that dynamin and actin recruitment both peaked at scission and that the final burst of dynamin recruitment lagged the onset of actin polymerization by ∼20 s (Figure 4C).

### Sequential Recruitment of Positive and Negative Regulators of Actin Polymerization

It is generally accepted that actin polymerization plays a role in some (but not all, see [52],[53]) forms of CME [9],[11],[18],[44],[46],[47],[50],[54]. Here, a more accurate measurement of actin dynamics using the ppH assay revealed an ordered sequence of proteins involved in actin dynamics. After the Arp2/3 complex activator N-WASP, which peaked before all the other actin module proteins and groups with the dynamin module, the F-actin-binding proteins Arp3, Abp1, cortactin, and lifeAct were recruited (Figure 4D). Unique among tested proteins, the average lifeAct signal was significantly below random prior to scission (Figure 4A), probably because bright stress fibres adjacent to sites of scission artificially lowered the background subtracted fluorescence value (e.g., see Figure S4).

Peak recruitment of the actin-severing protein cofilin [55] and the Arp2/3 suppressor coronin [56] were both significantly skewed post-scission, suggesting an ordered shut down of the actin polymerization machinery and disassembly of scission-associated actin (Figure 4D).

### Sequential Recruitment of Curvature-Generating/Sensing BAR and F-BAR Domain Proteins

Based on contemporary models of CME [57] we predicted that recruitment of BAR and F-BAR domain proteins should follow patterns consistent with the differing curvatures of their respective membrane-binding domains, since purified proteins induce different degrees of curvature in membrane tubulation assays in vitro and membrane curvature increases as CCSs invaginate [6],[7]. The sequential recruitment of the F-BAR domain protein syndapin2 and a group of NBAR domain proteins (endophilin2, BIN1, and amphiphysin1) followed by scission matched this prediction (Figure 4E). Similar to syndapin2, NBAR proteins were also rapidly discarded following scission, presumably because of the collapse of the highly curved membrane neck at the moment of scission. However, the recruitment of the BAR domain protein SNX9 differed from prediction. SNX9 recruitment began before scission, peaking ∼12 s after scission, similar to coronin and cofilin rather than to its binding partner, dynamin (Figure 4E). Similarly, the recruitment of the F-BAR domain proteins CIP4 and FBP17 also differed from prediction [6] (Figure 4F). Both proteins showed complex recruitment dynamics, with components of recruitment both before and after scission and, strikingly, FBP17 recruitment peaked markedly post-scission, at a time similar to that of GAK (Figure 4F).

### The Invariant Recruitment of GAK

It was shown previously that the kinase GAK, which is necessary for CCV uncoating, was recruited shortly after the large GTPase dynamin to sites of CME [15],[16]. Here, we found that GAK recruitment commenced at scission and peaked on average ∼8 s thereafter, as predicted. The recruitment profile of GAK was the same for both terminal and non-terminal scission events (Figure 4G). In the canonical model of CME, bona fide endocytic structures were represented as spot-like CCSs that formed de novo [23]. We therefore analysed a subset of 100 scission events associated with spot-like CCSs that formed de novo and found that the first detected scission event occurred, on average, 93 s following CCS inception (minimum = 20 s) and similar to the 100 s calculated in a previous study [10]. The GAK recruitment signature was again similar (Figure 4G, inset), and therefore, irrespective of the behaviour of the host CCS, the dynamics of the uncoating reaction associated with scission events were comparable.

The recruitment signature of GAK defined a module including ACK1, a serine threonine kinase implicated in tumorigenesis [58] and OCRL1, a 5′ phosphatase and Rab5a effector [59]. Interestingly, GAK and OCRL1 were recruited only after scission, whereas ACK1 was gradually recruited as CCSs matured (Figure 4A). The last module to be recruited consisted of the Rab5a effector APPL1 [60] and Rab5a itself (Figure 4A). The Rab5a signal was small and temporally spread, but significantly raised above baseline. Most likely this marks the outer limits of recruitment detection using the ppH protocol.

Having accurately measured the recruitment signatures of a representative set of endocytic proteins we next asked whether the same set of proteins was recruited to scission events at different dynamic classes of CCSs.

### The Relationship between the Dynamic Characteristics of CCSs and Patterns of Protein Recruitment to Sites of Scission

Previous studies defined different populations of CCSs on the basis of size (i.e., spot-like CCSs versus larger CCSs) and lifetime or whether CCSs disappeared following scission (terminal events) or persisted (non-terminal events) [10],[23],[28],[61]. Detailed mechanistic inferences have been based on these types of dynamic classification [23]. Therefore, we explored whether the set of endocytic proteins recruited differed between terminal and non-terminal scission events or between scission events at CCSs of different size or lifetime.

### Patterns of Protein Recruitment to Terminal or Non-Terminal Scission Events

First we analysed whether the same set of proteins was recruited to scission events at terminal and non-terminal scission events. Terminal and non terminal events were sorted by computing the ratio of average *F*_TfR7_ before and after scission (see Materials and Methods). For all constructs tested, there was approximately the same number of events in each category (Table S1). For all proteins tested the average fluorescence profiles were strikingly similar between terminal and non-terminal events before scission, with occasional shifts towards higher values for non-terminal events (Figure S8). This strongly suggests that the mechanisms of protein recruitment were the same for both classes of events. By contrast, the recruitment signatures after scission differed markedly for proteins that were significantly recruited at time points well removed from scission such as clathrin module proteins or some dynamin/myosin module proteins. Interestingly, in many recruitment signatures (e.g., Eps15, mu2, myosin6, or CALM), the average fluorescence trace of non-terminal events increased steadily after scission to a maximum around 40 s post-scission, suggesting a characteristic time course of CCS maturation between successive scission events, and similar to findings in a previous study [10].

### Patterns of Protein Recruitment to Scission Events Hosted by CCSs of Different Size or Lifetimes

**Figure 5:**
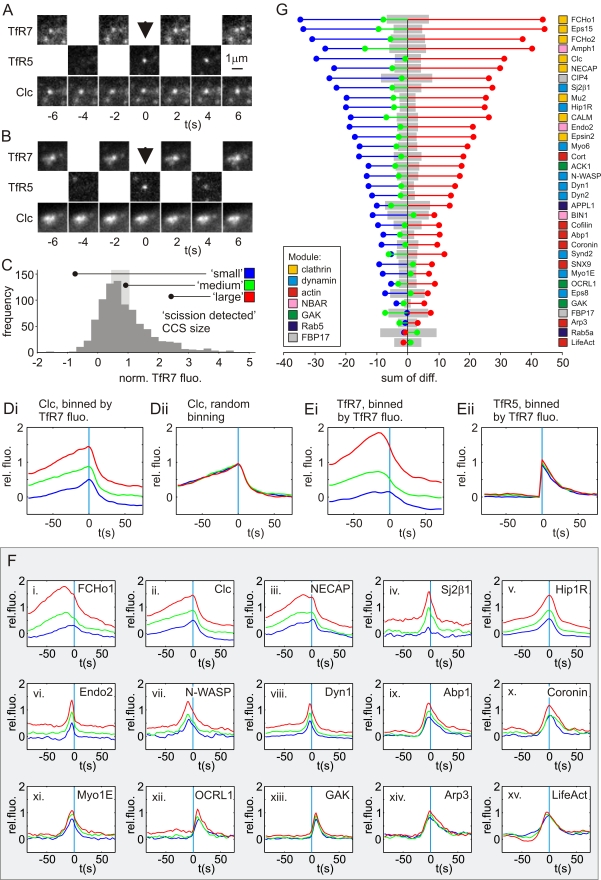
Scaling relationships between CCS size and recruitment signatures. (A–E) Analysis of cells coexpressing Clc-mCherry and TfR-phl. (A) Example scission event at a punctate CCS. (B) Example scission event at a larger CCS. The larger (brighter) CCS was associated with a larger (brighter) patch of TfR-phl at pH 7.4. (C) Histogram of normalised TfR7 fluorescence (*F*_TfR7_), averaged over −18 to −10 s preceding scission. Size classes defined as indicated: blue = {*F*_TfR7_ <33^rd^ percentile}; green = {33^rd^ percentile <* F*_TfR7 _<66^th^ percentile}; red = {*F*_TfR7_ >66^th^ percentile}. (Di) Average normalised *F*_Clc_ signatures for classes defined in (C). The signatures are well separated, indicating that *F*_Clc_ scaled with *F*_TfR7_. (Dii) Average normalised Clc-mCherry fluorescence signatures (*F*_Clc7_) for randomly allocated classes. Note the signatures are very similar. (Ei) Average normalised *F*_TfR7_ signatures for classes defined in (C). The signatures are well separated, as expected. (Eii) Average normalised *F*_TfR5_ signatures for classes defined in (C). The signatures are very similar, indicating that TfR5 fluorescence did not scale strongly with CCS size. (F) Example traces showing the scaling relationship between *F*_TfR7_ and the respective RFP recruitment signatures. (G) Stem plot of sum of differences between class averages and the overall average ordered by magnitude, colour coding as in (B). Grey bars indicate the 95% confidence interval for random event assignment to classes.

We established that there was a good correlation between Clc-mCherry fluorescence and TfR7 fluorescence and that, by inference, TfR7 fluorescence could be used to confidently predict the relative size or lifetime of CCSs (Figures 1E, S3, 5A, and 5B). Therefore, to investigate the relationship between CCS size and patterns of protein recruitment, TfR7 patch fluorescence was normalised by cell, and, for each trace, the average fluorescence (*F*_TfR7_) was calculated over the time interval −18 s to −10 s relative to scission (Figure 5C). For each cell the *F*_TfR7_ values formed a continuous distribution (Figure 5C) that was divided into three equally populated groups representing “small” CCSs (blue fluorescence traces), “medium” CCSs (green fluorescence traces), and “large” CCSs (red fluorescence traces, Figure 5). As expected, when the normalised fluorescence recruitment traces for Clc-mCherry were assigned to CCS size groups 1–3, the group average recruitment signatures were well separated (Figure 5Di). This simply reflected the fact that larger CCSs had more clathrin and confirmed that TfR7 fluorescence could be used to predict CCS size (see also Figure S3). However, and as a control, when Clc-mCherry recruitment traces were randomly assigned to three groups, the average traces for groups 1–3 were almost identical (Figure 5Dii). Thus, we can be confident that Clc-mCherry fluorescence scaled strongly with TfR7 fluorescence, as expected. Similarly, when TfR7 fluorescence traces were assigned to CCS size groups 1–3, the fluorescence signatures were (by definition) well separated (Figure 5Ei), but when TfR5 fluorescence traces were assigned to CCS size groups 1–3 and averaged, the TfR5 class averages were virtually identical (Figure 5Eii). Therefore (and similar to Figure S3), the amount of TfR internalized did not scale strongly with the size of the host CCS, consistent with the idea that quantized scission events occurred at CCSs of apparently different sizes.

The analysis was repeated for the 34 endocytic proteins of this study to assess how different recruitment signatures scaled with CCS size. Sample classified recruitment signatures are shown in Figure 5F, and, for each protein, the relative strength of the scaling relationship between CCS size and protein recruitment was visualised by calculating the summed absolute difference between the group averages and overall average (Figure 5G, 95% bootstrapped confidence interval in grey). Thus, for example, the average FCHo1 fluorescence traces for the small and large groups of CCSs (Figure 5G, blue and red, respectively) were well separated from the pooled FCHo1 average fluorescence trace, indicating a strong scaling relationship between CCS size and the amount of FCHo1 at the CCS. The scaling relationship was significant because it exceeded the boundaries of the confidence interval in grey (Figure 5G).

In general, the group averages for structural proteins such as clathrin and the adaptor proteins (mu2, Eps15, and FCHo1/2) scaled strongly with CCS size. The relationship between Dyn1-mCherry recruitment and TfR7 cluster size was more complex. As noted earlier, low amplitude flickering of Dyn1-mCherry was noted at CCSs before the final recruitment burst that marked scission (Figures 2B, 2C, 3A, and 3B). The overall amplitude of Dyn1-mCherry recruitment did scale with CCS size, but this could be explained by the difference in offset of the “pre-scission” signal, consistent with two components to the Dyn1-mCherry signal: pre-scission recruitment scaled with CCS size, suggesting a link with clathrin in the host CCS, but the burst of dynamin associated with scission was of relatively constant amplitude, consistent with recruitment to budding structure of constant dimensions. Other transiently recruited proteins, such as endophilin2, showed similar behaviour (Figure 5F). A notable exception was synaptojanin2β1, which showed robust recruitment to large CCSs but lower amplitude recruitment to smaller CCSs (Figure 5F). Finally, the amplitudes of Arp3 and lifeAct recruitment signatures were independent of CCS size (Figure 5F and 5G). In general, proteins of the actin module were among the proteins least dependent on the size of the host CCS.

A second characteristic that has been used to define dynamic groups of CCSs is lifetime [27],[28]. To test whether TfR7 patches could be used as indicators of CCS lifetime, TfR7 patches from cells expressing Clc-mCherry were tracked, and the set of 11,091 track histories was classified according to the presence or absence of scission events. The estimate of scission-undetected TfR7 patch lifetime was 33.8 s, which was ∼11% lower than the 38 s estimated using Clc-mCherry as a marker for CCSs. This slightly lower lifetime is because the TfR-phl signal tended to drop slightly before the Clc-mCherry signal in the run-up to scission (Figures 2D and 4B). The estimate of scission-detected TfR7 patch lifetime was found to be 178 s, which is within 6% of the 189 s estimated using Clc-mRFP as a CCS marker. Therefore, TfR7 patch lifetime could be used to estimate CCS lifetime.

**Figure 6:**
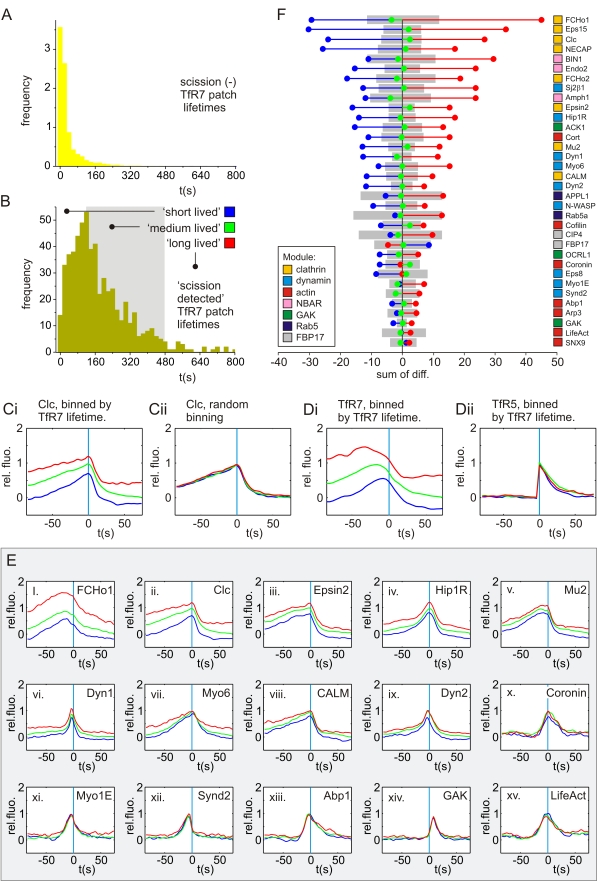
Scaling relationships between CCS lifetime and recruitment signatures. (A and B) Histograms of TfR7 patch lifetime (LT_TfR7_) for scission undetected (A) and scission detected (B) TfR7 patches in cells coexpressing Clc-mCherry and TfR-phl. (B) Time classes indicated in grey: blue = {LT_TfR7_ <120 s}; green = {120 s < LT_TfR7_ <480 s}; red = {LT_TfR7_ >480 s}. (Ci) Average, normalised *F*_Clc_ traces for the three classes defined in (B). The *F*_Clc7_ class averages are well separated, indicating that *F*_Clc7_ scaled with LT_TfR7_. (Cii) Average, normalised *F*_Clc_ traces for three randomly allocated classes. Note the *F*_Clc7_ traces are very similar. (Di) Average, normalised *F*_TfR7_ traces for the three classes defined in (B). The traces are well separated, indicating that *F*_TfR7_ scaled with LT_TfR7_. (Dii) Average, normalised *F*_TfR5_ traces for the three classes defined in (B). The traces are very similar, indicating that *F*_TfR5_ did not scale strongly with LT_TfR7_. (E) Example traces showing the scaling relationship between LT_TfR7_ and the respective RFP recruitment signatures. (F) Stem plot of sum of differences between class averages and the overall average ordered by magnitude, colour coding as in (B). Grey bars indicate the 95% confidence interval for random event assignment to classes.

Similar to CCS size, scaling relationships were found between CCS lifetime and the relative amount of protein recruited (Figure 6A–6E). Longer lived CCSs tended to have more clathrin and adaptor proteins while, by contrast, GAK and lifeAct showed the weakest dependence on CCS lifetime (Figure 6E and 6F). This is trivially explained if larger CCSs tended to have longer lifetimes, and indeed TfR7 patch fluorescence and lifetime had a positive (though modest) correlation of 0.29 (*p*<0.05, full set of events used), similar to previous observations [62].

Collectively, these analyses demonstrate that the same set of proteins was recruited to scission events at different dynamic groups of CCSs, with subtle scaling relationships between CCS size, lifetime, and the relative amount of different proteins recruited. However, this analysis did not reveal whether the same set of proteins was recruited to each scission event.

### Mechanistic Heterogeneity among Scission Events

The physical properties of CCSs were not predictive of which endocytic proteins were recruited to scission events (Figures 5 and 6). However, there is evidence that CCSs with different complements of adaptor proteins and receptor cargo coexist in the same cell [63], and it has been shown that the dependence of CME on actin differs between the apical and basolateral domains in epithelial cells [53]. Therefore, there may be differences in the set of proteins recruited to individual scission events, even though they internalized similar amounts of the same cargo (TfnR-phl).

First, we checked whether the automated selection criteria were biased towards a mechanistically distinct subtype of CME. For five example cells expressing mCherry chimeras of Clc, Hip1R, N-WASP, dynamin1, or GAK we visually inspected the set of events rejected by our selection criteria and “recalled” events judged to be bona fide by a human operator (see Materials and Methods; Figure S10). There was no significant difference in the kinetics of protein recruitment to the subset of “recalled” events when compared to events automatically selected (Figure S10). Therefore, no measurable bias was introduced by the parameters set for automatic detection.

**Figure 7:**
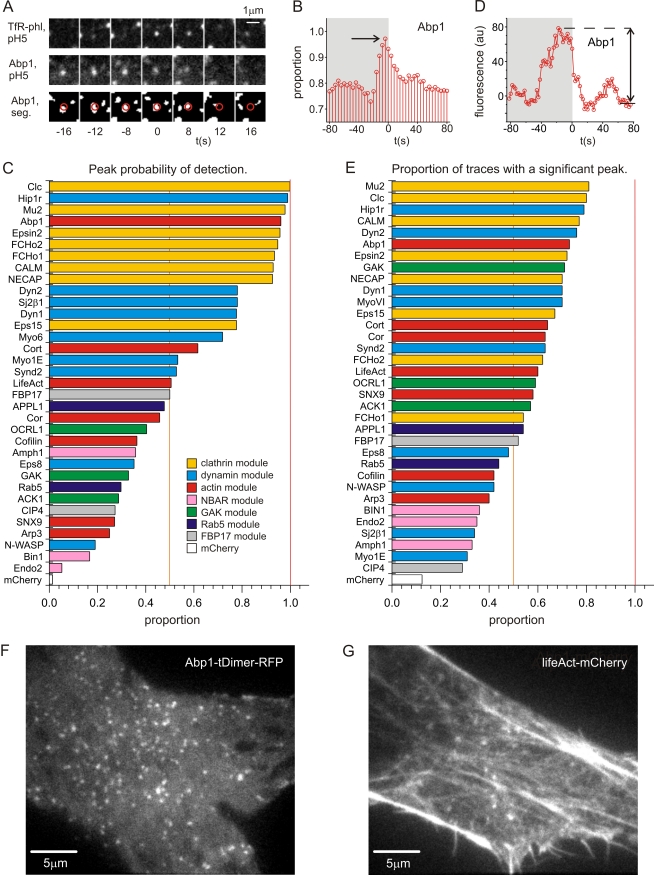
The probability of detecting protein recruitment to sites of scission. (A) A scission event (upper panel) defined a spot on the plasma membrane to which Abp1-mCherry (middle panel) was recruited. The recruited Abp1-mCherry was identified as an “object” in segmented images (lower panel). A region of interest (red circle) centred on the scission event (upper panel) was interrogated at each successive frame in the 80 s before and after scission to find whether a segmented object of more than three pixels and >8 s dwell time was present at the site of scission or not. Frames in which an object was detected were scored “1”, and “0” otherwise. The filtering thresholds were set using mCherry as a negative control, which scored a peak detection probability of 0.013, wherein residual detection of mCherry “objects” was due to detector noise. (B) The analysis was repeated for all events and the average scores calculated to yield a time-resolved profile representing the “probability of object detection” at a given frame relative to scission. (C) The analysis was repeated for all the tagged endocytic proteins, which were ranked by probability of detection and colour coded according to the module membership (as defined in Figure 4). (D) In the second analysis strategy, fluorescence traces, such as this example fluorescence trace for Abp1, were analysed to identify peaks (defined as biggest peak greater than six standard deviations of the last six *F*_RFP_ values of the recording), and the proportion of scission events with a “significant peak” of recruitment was determined. (E) The analysis was repeated for all the tagged endocytic proteins analysed, which were subsequently ranked. (F and G) Example cells expressing Abp1-mCherry (F) or lifeAct-mCherry (G) illustrating the different patterns of fluorescence.

Second, we determined how many scission events scored positive for recruitment of any given protein (Figure 7). The probability of detecting protein recruitment is dependent on multiple physical factors including signal and detector limitations, the kinetics of protein recruitment, and the magnitude and texture of background fluorescence (see Materials and Methods). Two strategies were used to detect recruitment (Figure 7). The first strategy was biased towards the detection of proteins recruited with slower kinetics and used image segmentation to determine the maximum probability of detection relative to scission (Figure 7A–7C). The second strategy was biased towards the detection of more transient signals and identified significant peaks in the quantified fluorescence traces (Figure 7D and 7E).

Of the 34 proteins analysed 25 proteins from six modules (clathrin, actin, dynamin, GAK, FBP17, and Rab5 modules) were detected at more than 50% of scission events using either detection strategy (Figure 7). It seems unlikely that these 25 proteins were recruited to distinct and mutually exclusive variants of CME, and there was most probably some overlap between any given pair. Of these proteins high-abundance structural proteins such as clathrin, adaptor proteins, and other members of the clathrin module were most readily detected (gold bars, Figure 7C and 7E). Proteins of the dynamin module were the next most frequently detected (pale blue bars, Figure 7C and 7E). Proteins of the actin module (red bars, Figure 7C and 7E) were detected less frequently, with the notable exception of Abp1 (maximum probability of detection = 0.97, Figure 7C). The clathrin- and F-actin-binding protein Hip1R was also detected with high frequency (maximum probability of detection = 0.99, Figure 7C). Detection of the F-actin-binding protein Abp1 was facilitated by the proteins' punctate distribution and low background fluorescence (Figure 7F). By contrast, an alternative F-actin marker, lifeAct, was recruited promiscuously to all F-actin structures at the cell cortex, which gave a bright and highly textured background, and most likely contributed to the lower probability of detecting lifeAct at scission events (Figure 7G).

The set of proteins that were detected at ∼50% of scission events or fewer using either detection strategy included the NBAR module (BIN1, Endo2, and Amph1, pink bars, Figure 7C and 7E). However, because NBAR proteins are thought to be essential components of the CME machinery [9],[54], this most likely represents limitations of detection, as found previously [37], rather than core mechanistic differences between scission events. The low incidence of detection of other proteins is less easy to interpret. For instance, CIP4 and Rab5 were detected with low incidence, but the significance of this currently remains unclear (Figure 7).

## Discussion

### The Properties of Endocytically Active CCSs

Early EM studies revealed clathrin-coated invaginations at the substrate proximal surface of adherent cells as discrete entities, in clusters or at the edges of large, flat lattices of clathrin [25],[26]. Subsequent live-cell imaging studies using TIR-FM described, for a variety of cell types, corresponding heterogeneity among CCSs labelled with clathrin-FP at the substrate proximal surface of adherent cells [3],[10],[23],[28],[31],[62]. It was shown that both transient spot-like CCSs (average lifetime = ∼40–60 s) and larger, longer lived CCSs (average lifetime = ∼60 s to 10 min [or more]) coexisted in NIH-3T3 fibroblasts, HeLa, and COS cells [10],[23], while transient spot-like CCSs (average lifetime = ∼40 s) predominated in freshly plated BSC1 cells [3],[28],[31],[62]. Larger and longer lived CCSs were triggered by specific receptor/adaptor combinations [62], and cell adhesion could also play a role [22]. Faced with such natural ultrastructural and dynamic heterogeneity, it was important to establish which CCS characteristics, measured in live-cell TIR-FM experiments, defined CCS intermediates in CME. The detection of individual scission events presented here and previously [10] helps achieve this by quantifying the relationships between scission and CCS dynamics and size in an unbiased manner.

We can make four main conclusions from our study of CCS characteristics relative to scission. First, the lifetimes of scission-detected CCSs followed a left-skewed distribution ranging from a few tens of seconds through to hundreds of seconds, as predicted by earlier studies [3],[28]. The shorter lived population of scission-undetected CCSs identified most likely corresponded to abortive CCSs described previously [3],[28], although intracellular CCSs may have contributed. The average time between CCS inception and the first detected scission event was ∼100 s (minimum lifetime of 20 s), which reflected the time required to construct a productive CCS. However, CCS lifetimes should be interpreted with caution since CCSs can host multiple scission events (see also [10] and the third point below).

Second, the size of scission-detected CCSs followed a left-skewed distribution without obvious quantization. No correlation was detected between overall CCS size and the amount of TfR-phl cargo internalized by scission events, consistent with an earlier study [10].

Third, the disappearance of spot-like CCSs, which has been widely used as a fiducial marker for CME [18],[19], coincided with scission events with the predicted frequency but it was found to be an imprecise marker for scission (Δ*t* between scission and spot-like CCS disappearance = 7±22 s; Figures 1 and S2). Moreover, CCS disappearance did not report all scission events, and approximately ∼50% of scission events were classified as non-terminal because the host CCS did not completely disappear following scission. Indeed, CCSs could host multiple scission events before disappearing (see also [10]).

Fourth, evidence that the scission events detected at different dynamic groups of CCSs proceeded through to completion (i.e., CCV uncoating) was provided by the remarkable invariance of the GAK recruitment signature. The kinase GAK is an established marker for CCV uncoating [15],[16], and the GAK recruitment signature was the same for terminal and non-terminal scission events, for scission events at spot-like CCSs that formed de novo, and for scission events at different size and lifetime classes of CCSs.

**Figure 8:**
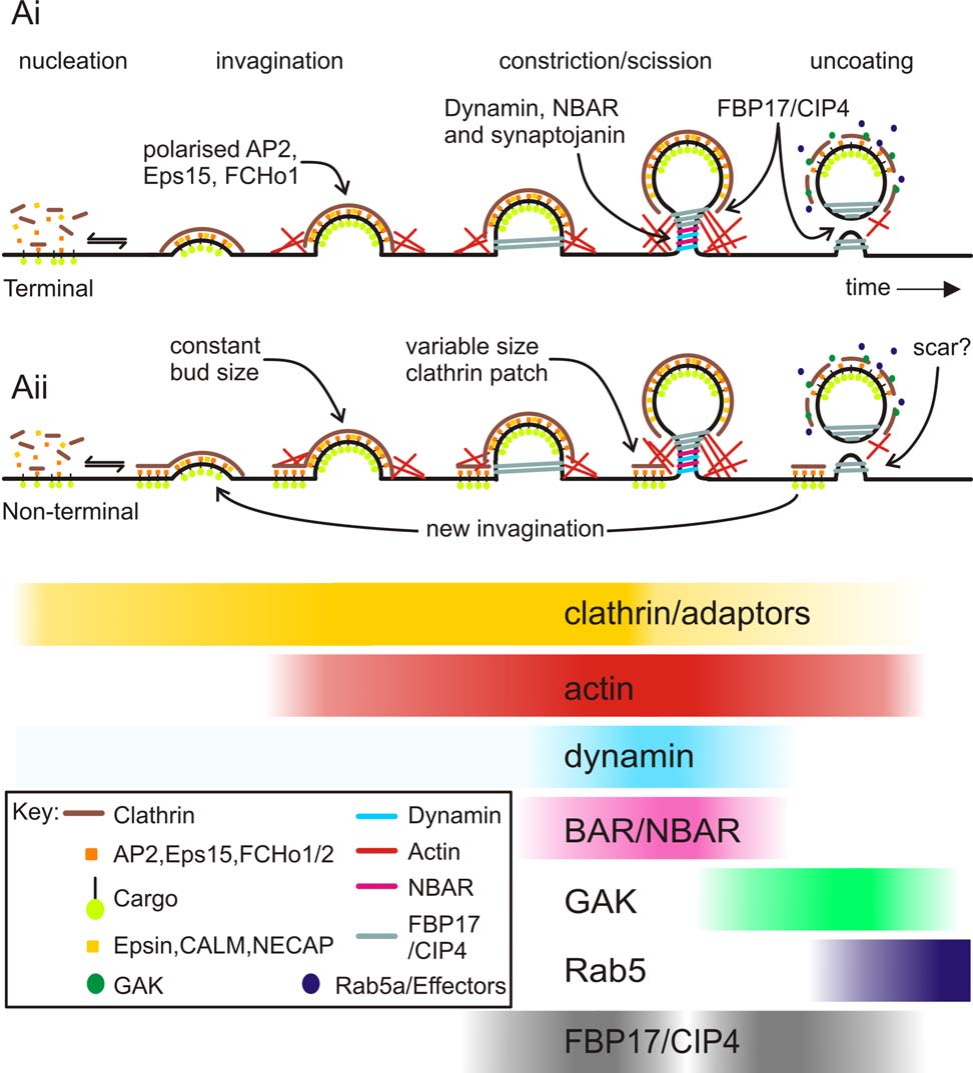
A simplified canonical model of mammalian CME. A simplified schematic illustrating the relative timing of recruitment of the seven different endocytic protein modules to sites of scission, highlighting some unexpected findings for future investigation. The patterns of recruitment are the same for terminal events (Ai) and non-terminal events (Aii). The heterogeneous size of endocytically productive CCSs is most easily explained if clathrin-coated buds formed at the edges of clathrin patches of variable size, thus accounting for the variability in fluorescence of endocytically active CCSs (Aii). Repeat scission events most likely occurred by re-growth of clathrin-coated invaginations at the edge of “host” patches of clathrin (lower curved arrow, [Aii]).

The most parsimonious explanation for these findings is that CCVs, of similar size, could either bud in isolation or from larger, heterogeneous CCSs (Figure 8). This is consistent with the relatively constant dimensions of clathrin-coated invaginations previously observed by EM, irrespective of whether the invaginations were isolated or part of larger CCSs [25],[26]. Based on these results we conclude that the classification of endocytically active CCSs, observed at optical resolution using TIR-FM, should be broad to encompass the heterogeneity of scission-competent CCS sizes and lifetimes. As a practical guide, any CCS that colocalizes with acid-accessible TfR-phl and that exists for more than 20 s could be considered scission competent [10] and potentially capable of hosting multiple scission events.

### The Same Core Machinery of CME Operates at Different Dynamic Classes of CCSs, but Subtle Variations in Mechanism May Occur

In a further exploration of the organization of CME in fibroblasts we analysed the recruitment of 34 types of endocytic protein to scission events, 30 of which were native to NIH-3T3 fibroblasts. To appreciate this analysis properly it is important to consider what physical factors contribute to the observed dynamics of protein recruitment and the resulting shapes of ensemble recruitment signatures. First, the fluorescence signals measured at single scission events using TIR-FM occur in a volume of ∼1 al, illuminated by an evanescent field in which the intensity of the electromagnetic field decreases exponentially as a function of distance in the *z*-axis [64]. Due to the small depth constant of the illuminating evanescent field (∼100 nm) and the comparable dimension of an invaginating CCP (∼100 nm diameter), for two proteins to show a similar average recruitment signature they must be recruited to the detection volume over a similar time course and must share a similar spatial distribution at the developing CCP as it projects into the evanescent field along the *z-*axis [10],[31]. Second, a recruitment signature reflects the average concentration of an FP-labelled protein at the site of endocytosis relative to the cytoplasm. Labelled protein, expressed at low levels, must compete with endogenous proteins for recruitment, and this, combined with detector limitations and the relatively low quantum efficiency of mCherry [65], most likely contributes to noise among individual recruitment profiles and influences the probability of detecting protein recruitment. We established that for one example protein, dynamin1, the noise appeared to be unstructured and that the trajectories of the averaged recruitment signatures for dynamin1 in NIH-3T3 cells were remarkably stable.

A detailed analysis suggested involvement of the core clathrin, actin, and dynamin modules in the majority of scission events since all coat components (clathrin, AP2, epsin2, FCHo, CALM, and NECAP) and both Hip1R (which binds clathrin and F-actin [44]) and Abp1 (which binds dynamin, F-actin, and Arp2/3 [66]) were detected at >90% of scission events, and dynamin1/2, synaptojanin2β1, myosin6, and Eps15 were detected at >75% of events. These findings agree with the widely accepted view that TfR internalizes via a clathrin- and dynamin-dependent pathway [67],[68] and are in agreement with earlier studies that demonstrated an important, though nonessential, role for actin in CME in fibroblasts ([9]–[11], but see [23]). The fact that other proteins such as the BAR domain proteins endophilin2 or BIN1 were detected at only a subset of scission events suggests that there were inherent limitations of recruitment detection, since these proteins are thought to be essential for scission [9],[54]. However, it remains possible that there were genuine molecular differences between scission events, perhaps through the influence of other types of (unlabelled) receptor cargo [63], in response to changes in physical parameters, such as membrane tension [69] or because of genuine underlying variability in the core mechanism of CME [53]. Nonetheless, and based on the data presented here, at optical resolution potential molecular differences between scission events in NIH-3T3 cells did not correlate with obvious differences in CCS behaviour.

Next we explored scaling relationships between CCS size and lifetime and the set of proteins recruited to scission events. As shown previously [62], CCS lifetime and size were moderately correlated, with longer lifetimes for larger CCSs, and, as predicted, the recruitment signatures of some proteins such as the coat protein clathrin and adaptor proteins scaled with overall CCS size. A set of core components (e.g., dynamin and endophilin2) showed more complex scaling relationships with CCS size, perhaps reflecting variable degrees of recruitment to the budding and non-budding portions of larger CCSs. However, the recruitment signatures of a core set of proteins including GAK (a kinase essential for the uncoating reaction [16],[70]), and most notably actin and actin-binding proteins, were independent of CCS size. This is consistent with our central thesis that CCVs of relatively constant size budded at host CCSs of diverse size and lifetime via a common core mechanism, and supports a role for actin in CME in NIH-3T3 fibroblasts [10],[11].

### Similar to Yeast, Mammalian CME Has a Modular Design

Seminal imaging studies from the Drubin lab and other groups revealed the modular organization of yeast endocytosis [24]. Here it was shown that at least four modules or groups of proteins showed similar recruitment dynamics to sites of endocytosis at yeast actin patches [24]. More recently, comparisons were drawn between the modular organization of yeast and mammalian endocytosis, with an emphasis on the conserved role of actin [9],[71]. However, earlier TIR-FM studies of the late stages of mammalian CME used the disappearance of spot-like CCSs as a fiducial marker, which could not sample endocytic events from all dynamic classes of CCSs nor yield a temporally precise estimate of scission. Consequently, the recruitment dynamics of endocytic proteins could only be broadly classified as “early” and “late” (Figure S1). The data presented here, based on the comparison of accurately measured recruitment signatures derived from large datasets (∼1,000 events), give a more detailed overview of the modular organization of mammalian CME. The modules identified here comprise the following (Figure 8): (1) the coat module, divided into (i) a clathrin sub-module (epsin2, CALM, clathrin light chain, and NECAP) and (ii) an adaptor/F-BAR sub-module (FCHo1/2, Eps15, AP2); (2) the NBAR domain module (endophilin2, amphiphysin2, and BIN1); (3) the actin module, divided into (i) actin polymerization sub-module (Abp1, cortactin, and Arp3) and (ii) actin depolymerization/suppression (cofilin, coronin1B, and SNX9); (4) the dynamin/myosin/N-WASP module (dynamin1, dynamin2, synaptojanin2β1, myosin1E, N-WASP, Eps8, Hip1R, myosin6, and syndapin2); (5) the GAK/post-scission module (GAK, ACK1, and OCRL1); (6) the Rab5a module (Rab5a and APPL1); and (7) the FBP17/CIP4 module, based on the unique recruitment signatures of these two proteins and dissimilarity to any other recruitment signatures.

The shapes and relative timing of many of the recruitment signatures are broadly consistent with measurements made in previous imaging studies in yeast [24] and in mammalian cells [6],[18],[19]. In addition, many recruitment signatures provided new information as a consequence of improved accuracy. First, and as predicted from a previous study [31], the recruitment signatures of members of the adaptor sub-module decreased before scission because of polarization in the developing invagination. In addition, the F-BAR domain proteins FCHo1 and FCHo2 showed similar recruitment signatures, suggesting these curvature-inducing proteins were also polarized and consistent with a proposed role for FCHo proteins in the early stages of the invagination process [43]. Second, it was predicted that actin recruitment should begin before dynamin recruitment at sites of scission, although time-locked measurements with the required accuracy to test this hypothesis had not previously been made [9],[18]. Here, we showed that the onset of actin polymerization did indeed precede the final burst of dynamin recruitment by ∼20 s, consistent with a role for actin polymerization early in the invagination stage of CME and the later recruitment of dynamin to the deeply invaginated CCP, where it executed scission [9] (Figure 8). We also discovered that coronin1B and cofilin, proteins involved in the down-regulation of actin polymerization and F-actin severing, respectively, were recruited at later time points, again similar to yeast endocytosis [24],[72],[73]. Third, it was proposed that scission of endocytic invaginations in yeast is triggered by a PI-phosphatase that dephosphorylates PiP2 and thus induces a line tension in the membrane neck [74]. In mammalian cells the large GTPase dynamin is thought to execute scission [9],[30], but, intriguingly, the recruitment of the PI-phosphatase synaptojaninβ1 showed a recruitment trajectory similar to that of dynamin (and proteins of the NBAR module) and also peaked at scission (Figure 4). Therefore, it is plausible that induction of a line tension also contributes to the mechanochemistry of scission in mammalian cells [74]. Finally, it was predicted that recruitment of F-BAR and BAR domain proteins should follow an ordered sequence dictated by their preference for different-curvature membrane tubules in vitro [75] and that recruitment should occur over a trajectory similar to that of actin polymerization [6],[9],[76]. The ordered recruitment of syndapin2 and the NBAR module (endophilin2, BIN1, and amphiphysin1) did indeed match this prediction. However the post-scission peak recruitment of SNX9 and the complex, biphasic recruitment of FBP17 and CIP4 did not. These findings illustrate that the recruitment sequence of these BAR and F-BAR domain proteins could not be predicted purely on the basis of either structural information or biochemical properties. The possible function(s) of SNX9 and FBP17/CIP4 post-scission remain to be elucidated, although it is possible that these proteins may act as relays to recruit additional binding partners to the newly formed endosome (Figure 8).

### Conclusion

The study presented here employed the detection of scission events to construct what is to our knowledge the highest resolution temporal map of mammalian CME to date. The map (1) suggests a simplified canonical model of mammalian CME in which the same core mechanism operates at both spot-like CCSs and larger CCSs observed with fluorescence microscopy, (2) illustrates the similar modular organization of mammalian and yeast endocytosis, and (3) proves that recruitment dynamics of endocytic proteins such as the F-BAR protein FBP17 and BAR domain protein SNX9 cannot always be predicted from biochemical or structural properties.

## Materials and Methods

### Cell Culture and Imaging

NIH-3T3 cells were cultured as described previously [10]. Cells were co-transfected using Lipofectamine 2000 (Invitrogen) with human transferrin receptor fused to super-ecliptic phluorin (hTfnR-phl [10]) and the relevant endocytic protein open reading frame (ORF) fused to a RFP. Freshly transfected cells were replated onto pre-cleaned number 1 borosilicate glass coverslips (VWR International) and imaged 24–48 h later as described previously [10].

### Cloning and Expression Plasmids

ORFs of endocytic proteins were amplified by PCR (Phusion PCR kit; Finnzyme) from IMAGE clones (Geneservice), or directly amplified from cDNA libraries (see Table S2 for details of primers and cDNA sources for the expression constructs used). Each pair of PCR primers was engineered with the appropriate 3′ and 5′ restriction sites for cloning and sequence for either a 9-, 12-, or 13-amino-acid linker between the target protein and FP, as described previously [65]. The amplified cDNAs were cloned into mammalian expression vectors in frame with a RFP (in the case of Hip1R, tDimer [65]; in the case of myosin1E, mApple [77]; and for all other proteins mCherry [78]; see Table S2) to generate either N- or C-terminal fusion proteins upon expression.

### RT-PCR

Primers were designed to PCR a ∼700-bp fragment that was specific to the protein isoforms used in this study. Total cell RNA was purified from NIH-3T3 cells using the RNAeasy Mini Kit (Qiagen). RT-PCR reactions were run using the OneStep RT-PCR kit from (Qiagen) using the manufacturer's protocol. The QIAxcel capillary gel electrophoresis system (Qiagen) was used to visualise RT-PCR products. Samples were run using the DNA screening cartridge using the AM420 run settings (5 kV sample injection voltage for 10 s, 5 kV separation voltage for 420 s; suitable for DNA concentrations of 10–100 ng/µl). A photomultiplier detector converted the emission signal into a gel image and an electropherogram that allows visualisation and quantification, respectively, of each PCR product. The Biocalculator software package (Qiagen) was used to analyse the peaks for each sample. Aligment marker of 50 bp to 1.5 kb was used to align run samples.

### Imaging and Perfusion

The TIR-FM and ppH perfusion system have been described previously [10].

### Transferrin Uptake Experiments

Cells were transfected with plasmid encoding TfR-phl and RFP-tagged endocytic protein and plated onto coverslips 24 h before imaging. Transfected cells were located and imaged using a spinning disk UltraVIEW ERS confocal (PerkinElmer) using a ×40/1.4 NA oil immersion PlanApo objective (Olympus). After acquiring an initial image (denoted *t* = 0 min) transferrin conjugated to Alexa 647 (Tfn-A647; Invitrogen) was added to the chamber at 10 mg/ml in 10 mM HEPES buffer saline solution (pH 7.4). After 30 min at room temperature the cells were washed twice in 10 mM MES (pH 4.0) to strip away surface-bound Tfn-A647 and returned to HEPES buffer saline (pH 7.4). The cells were then imaged to determine uptake of transferrin (image denoted as *t* = 30 min).

### Spot Segmentation, Detection of Protein Clusters (CCSs, TfR7 Clusters, and TfR5 Vesicles), and Particle Tracking

Movies of cells during the alternate pH protocol were divided in four parts, TfR-phl at pH 5.5 (TfR5 movie) and at pH 7.4 (TfR7 movie), and the RFP fusion protein at the two pH values (movies RFP5 and RFP7). To detect protein clusters (CCSs or TfR7 clusters as in Figure S4, CCVs in the TfR5 movies) images were subjected to segmentation based on wavelet transform (Multidimensional Image Analysis [MIA] add-on to Metamorph 6, written by V. Racine and J.-B. Sibarita, Curie Institute, Paris, France). The objects detected were then tracked using a simulated annealing algorithm [79] to identify endocytic events. The output of this tracking was a series of coordinates corresponding to the centre of mass of the objects, with unique identifiers (event numbers).

To determine the lifetimes of CCSs using either Clc7 or TfR7, a different tracking algorithm was used to account for transient breaks in track histories of 1–2 frames (i.e., gap closing was incorporated). The coordinate lists generated by MIA were reassigned in Matlab using a nearest-neighbour algorithm (“track.pro”, John C. Crocker and Eric R.Weeks, http://www.physics.emory.edu/~weeks/idl/index.html). For Clc data, independent track histories generated by MIA from Clc7 and Clc5 data were combined and reassigned, while for TfR only the TfR7 data were used. To verify tracking fidelity the reassigned tracks were overlaid on the original image series in Matlab and inspected visually. Although the tracking was perhaps not as robust as more recently published techniques [27], it was sufficiently robust to differentiate between long-lived CCSs and shorter lived CCSs (Figures 1 and 6).

### Screening of Scission Events

All the tracked objects in the TfR5 movies were screened to identify genuine endocytic events using routines programmed in Matlab 7.4 (Mathworks). To qualify as bona fide events each candidate event required a TfR5 vesicle (i) that persisted for at least three frames (i.e., 8 s) following appearance, (ii) that appeared at least 20 frames after the start of the movie, or 20 frames before its end, to ensure quantification of signals for 80 s before and after the vesicle's appearance, (iii) that appeared and remained at more than seven pixels (0.7 µm) from the edge of the image, to ensure proper quantification (see below), (iv) that appeared de novo, and was not produced by the fusion of two objects or the dissociation of an object into two, (v) that overlapped, on appearance, with a pre-existing cluster detected in the segmented TfR7 movie, (vi) whose fluorescence was bigger than a defined SNR of 5 wherein SNR = (*F*_0_−av)/std, where *F*_0_ is the fluorescence at time 0, and av and std are the average fluorescence and standard deviation, respectively, in the five frames before vesicle appearance, and (vii) that was close to maximal fluorescence at the time of appearance. We calculated the slope of the fluorescence change in the first three frames of vesicle appearance (Figure 1) and discarded the events where this slope was greater than 0.1, which corresponds to a 10% increase in fluorescence.

The purpose of this screening was not to detect all events in a recording, but to have stringent criteria to select automatically a large proportion of events that were genuine scission events, to test a large number of candidate proteins in a manageable analysis time. Among the events that occurred at suitable times and locations (criteria i–iv), only 18.5%±0.8% of events (*n* = 191 cells) passed the last three criteria (v–vii), for a total of 239±11 candidate events per cell. Nevertheless, some false-positive events remained, so we reviewed our dataset visually by watching each event individually (the portion of the TfR5 and RFP5 movies around the 0 frame, and an average of five frames of movie TfR7 before the event) to assess if there were tracking errors, poor signal, simultaneous events nearby, or other problems. On average, 82.3%±0.9% (*n* = 191 cells) of events were confirmed by this second, manual screen, for a total of 191±8 confirmed scission events per cell.

To check for bias in the screening procedure we performed a visual screen on all tracked objects for five cells, each transfected with different mCherry-tagged proteins (1,400±360 tracked objects per cell). Of the events rejected by the automated screen (1,100±318 objects), a total of 10.3%±2.2% were visually identified as genuine scission events (104±24 events). Importantly, when the fluorescence from these “recalled” events was quantified and averaged, the RFP recruitment signatures were the same as the signatures obtained from “semi-automatically selected” events (Figure S10). The sum of absolute differences between average fluorescence traces of semi-automatically selected and recalled events was not statistically significant. This shows that our semi-automated procedure did not select a particular category of scission events.

### Quantification of Protein Recruitment during Scission Events

Images in the green fluorescent protein (GFP) and RFP channels were acquired simultaneously with a Dual View (Optical Insights) beam-splitter that was adjusted with an image of beads that fluoresce in the two channels (yellow fluorescein carboxylate beads, 0.2-µm diameter, Invitrogen) to minimize distortion from one channel to another. However, small (0–5 pixels) shifts remained in the two channels that needed to be corrected digitally for optimal colocalization. We used a third-order polynomial spatial transform that interpolates between ten bead pairs to make the correction. When we quantified experimental data we did not transform the raw images (i.e., interpolate and reassign pixel fluorescence values) but instead used the spatial transform to recalculate the vesicle centre coordinates in the RFP channel. This works well, since the difference between the coordinates of a pixel (*x,y*) in the green channel and its transformed coordinates (*u,v*) in the red channel is only ever a fraction of a pixel.

We quantified the fluorescence 20 frames before and 20 frames after the appearance of a vesicle for all four movies in a three-pixel-radius circle centred on the object coordinates at the time of appearance (frame 0) for this frame and the 20 preceding frames, then centred at the tracked vesicle coordinates during tracking, and then on the last known coordinates after tracking was lost. Local background was estimated in an annulus (three pixels inner radius, six pixels outer radius) by taking an average of pixel values between the 20^th^ and 80^th^ percentiles to avoid contributions from neighbouring brightly fluorescent patches. This quantification is similar up to this point to other quantifications performed by us in previous studies [10],[18].

To correct for bleed through from the GFP to RFP channels we introduced a bleed-through coefficient (BT) for each cell to correct the fluorescence values with the formula *F*_RFPx,corr_ = *F*_RFPx_−BT·*F*_TfRx_, where x is 5 or 7. Such corrections are acceptable as they involve only linear combinations of fluorescence values. BT was determined for each cell by minimizing the summed squared difference for values of BT taken between 0 and 0.05 in 0.001 increments (Figure S4). Values of BT were on average 3.00%±0.07% (*n* = 191). Differences in BT values could arise from small differences in background fluorescence, non-linearity in the camera, or changes over months of the optical properties of the various parts of the system (filters, mirrors, or camera). With this correction, fluorescence values from RFP5 and RFP7 could be combined to achieve a time resolution of 2 s (Figure S4).

To determine when the recruitment of a labelled protein became significant, we generated randomized datasets by shifting the event coordinates in a random manner within the cell footprint (Figure S4), and calculated fluorescence for all four movies as described above. We generated 200 randomized datasets for each cell, and then combined the average fluorescence measures to determine, for each data point, 95% upper and lower intervals (Figure S4).

To sort events into terminal and non-terminal events, we measured the average *F*_TfR7_ for four frames before scission and nine frames (36 s) after scission. The ratio between these two values was used to determine whether the event was terminal (ratio <0.4) or non-terminal (ratio >0.6). Events with ratios close to 0.5 were not sorted. To determine the time of peak RFP recruitment, we estimated a noise level with standard deviation of the last six *F*_RFP_ values (12 s) of the recording. If the maximum is bigger than a threshold (six times noise above average), the time of the maximum *F*_RFP_ value is taken as the maximum RFP recruitment time and used to construct the histograms in Figure 1F and others. The proportion of events with significant peak recruitment is given in Table S2 for each tested protein.

### Construction of Cloud Plot

The goal was to visualise the overall structure of the Dyn1-mCherry set of fluorescence recruitment traces and determine whether there were “natural” (as opposed to analyst-imposed) classes. First the amplitudes of fluorescence traces were normalised by cell over the range [0,1], and the mean of each fluorescence trace was subtracted to reduce dispersion in the *y*-axis. Each normalised, offset fluorescence trace was projected into an image matrix, and at those points where the fluorescence trace overlaid a pixel a “1” was added to the pixel value, “0” otherwise. The resulting density plot was log-transformed to visualise both high- and low-density features.

### Cluster Analysis of Protein Recruitment Signatures

We compared the average recruitment signatures by computing the correlation coefficients for each pair of curves corr(RFP*_a_*,RFP*_b_*). Correlation coefficients were 0.45±0.43 (average ± standard deviation, *n* = 561). We then used the correlation distance, dist(RFP*_a_*,RFP*_b_*) = 1−corr(RFP*_a_*,RFP*_b_*), to perform a hierarchical clustering using an average linkage algorithm that generated the dendrogram in Figure 4. This hierarchical cluster tree reflected the actual correlations between RFP curves, with a correlation coefficient between the cophenetic distances (the distances represented as horizontal bars in the tree) and the correlation distances of 0.81. Other linkage algorithms yielded lower correlation coefficients.

To perform these comparisons, we used the full range of measurements, from −82 s to +76 s relative to the time of vesicle detection. Away from time 0, the differences between the curves would be less significant than close to the moment of vesicle formation and so similarity measurements could be affected by the choice of time interval around vesicle creation. We performed the same clustering procedure using RFP measures only between −44 and +36 s relative to vesicle formation. The cluster tree generated was very similar to the one shown in Figure 4. There were only three minor differences between these two trees: (i) N-WASP grouped first with syndapin instead of with Eps8, (ii) dynamin2 grouped first with dynamin1 instead of with Hip1R, and (iii) coronin grouped first with Arp3 and cortactin instead of with SNX9 and cofilin.

Finally, for many proteins the non-terminal fluorescence traces showed little variation before and after scission (Figure S8, see definition below of these two types of events). The clustering could be different in an analysis using only the terminal fluorescence traces, wherein most proteins reach random values 80 s after scission. Therefore, we performed the clustering on non-terminal events only. Again, the resulting dendrogram was very similar to the one shown in Figure 4, with the same number of modules defined by a distance threshold of 0.2, and only minor differences: (i) ACK1 leaves the GAK cluster to be weakly (distance >0.2) attached to the dynamin cluster, (ii) endophilin groups first with syndapin within the dynamin cluster, and (iii) four other different groupings occur between proteins within the same cluster.

Overall, these tests suggest that the clusters defined in Figure 2 correspond to genuine similarities between the different RFP recruitment signatures that would correspond to functional units involved in CCV formation.

### Analysis of CCP Disappearance Events

To explore the relationship between scission and CCS disappearance NIH-3T3 cells were transfected with Clc-mCherry and TfR-phl and assayed using the ppH protocol. All CCSs were tracked as described above. The end of each track history was extended by 20 frames (40 s) by padding with the last detected CCS location, and the Clc-mCherry fluorescence and TfR5 fluorescence were quantified for each candidate CCS. To identify CCS disappearance, abrupt drops in Clc-mCherry fluorescence were detected by convolving each Clc-mCherry fluorescence trace with a one-dimensional kernel appropriately tuned for negative edge detection (a negative step function kernel, convolved with a Gaussian, σ = 36 s). Step decreases in Clc-mCherry fluorescence manifest as spikes in the convolved signal, and the maximum response was used to define a *t*_0_ for each CCP fluorescence trace. By definition, this algorithm aligns the Clc-mCherry fluorescence traces to their respective maximal negative derivatives (i.e., maximal rate of fluorescence decrease). Although this differs slightly to the algorithm used previously [18], the temporal alignment is more robust. The resulting candidate CCS disappearance events were screened by comparing the average fluorescence of the first nine time points (*t* = −80 s to *t* = −44 s) and the last nine time points (*t* = 44 s to *t* = 80 s) of the fluorescence trace. Only those traces showing a decrease in average fluorescence with a magnitude at least 2.5-fold greater than the standard deviation of the first nine values were deemed bona fide. This removed false-positive disappearance events (i.e., abrupt but incomplete drops in Clc-mCherry fluorescence). To detect scission events associated with disappearing CCSs the TfR5 trace associated with each candidate CCP was screened for step increases in fluorescence of at least 25 fluorescence units between a given time point *t* and the average fluorescence over of the previous four time points. This is a less stringent criterion for detecting scission events than used in the main analysis but it was less prone to discarding dim or noisy scission events. Of 197 disappearing CCSs, 107 (54%) were associated with a scission event (Figure 1), close to the prediction that50% of events would be detected when the cell is bathed in pH 7.4 solution, the other 50% being invisible as they occur when the cell is under pH 5.5 solution.

### Thin Section EM

NIH-3T3 cells expressing hTfR-SEpHl were isolated by FACS 24 h post-transfection, replated, and allowed to adhere overnight. To examine potential effects of acidic buffer on CCS morphology NIH-3T3 cells were incubated with MES buffered saline (pH 5.5) for 1 or 10 min before being washed briefly in PBS and fixed at room temperature in a solution of paraformaldehyde (2%) and glutaraldehyde (2.5%) in sodium cacodylate (0.1 M at pH 7.2). Fixed cells were harvested by scraping and centrifuged in a horizontal rotor (1,000 *g*, 5 min). The resulting cell pellet was placed in fresh fixative and stored at 4°C. In preparation for EM, fixed samples were washed thoroughly in sodium cacodylate buffer (0.1 M), post-fixed in OsO_4_ (1% in 0.1 M sodium cacodylate) for 1 h, and then washed with distilled water. Samples were stained en bloc with uranyl acetate (2%) in ethanol (30%) before dehydration in a graded ethanol series followed by 1,2-epoxypropane (propylene oxide) and then infiltrated and embedded in CY212 resin (Agar Scientific). Ultrathin (50–70 nm) sections were cut on a Reichert Ultracut E microtome and collected on uncoated 200-mesh grids. Sections were post-stained with saturated uranyl acetate before staining with Reynolds lead citrate. Images were acquired using a Philips EM208 microscope, with an operating voltage of 80 kV, and a CCD camera.

### Protein Structures

Graphics for protein structures were downloaded from the Research Collaboratory for Structural Bioinformatics (RCSB) consortium Protein Data Bank (PDB) website, where the original citations are also listed.

- Endophilin: http://www.rcsb.org/pdb/explore/explore.do?structureId=2C08
- Amphiphysin: http://www.rcsb.org/pdb/explore/explore.do?structureId=1URU
- APPL: http://www.rcsb.org/pdb/explore/explore.do?structureId=2Q12
- SNX9: http://www.rcsb.org/pdb/explore/explore.do?structureId=3DYT
- Syndapin: http://www.rcsb.org/pdb/explore/explore.do?structureId=3HAI
- FCHo2: http://www.rcsb.org/pdb/explore/explore.do?structureId=2V0O

## Supporting Information

Figure S1 **The canonical model of CME.** (A) The formation of a CCV—as deduced from EM, genetic, biochemical, and live-cell fluorescent imaging data—begins with the random nucleation of a small patch of adaptors, the F-BAR domain protein FCHo, and clathrin at the plasma membrane that is stabilised (possibly by the acquisition of cargo [3],[28]) to form a CCP. The CCP invaginates and grows by the addition of adaptors and clathrin triskelion before the large GTPase dynamin constricts and pinches off the membrane neck, releasing a CCV. Following scission, the coated vesicle is stripped of the clathrin coat by the ATPase Hsc70 and cofactors and is subsequently processed by the endosomal machinery. (B) A table summarising the findings of live-cell imaging studies of endocytic protein dynamics in mammalian cells. In dual colour imaging experiments, different reference signals have been used to align and decipher the recruitment dynamics of fluorescently tagged endocytic proteins to sites of endocytosis. In a typical experiment the disappearance or increase in mobility of CCPs labelled with GFP or RFP clathrin light chain was used as an indicator for endocytosis. This approach was used to characterise the recruitment dynamics of AP2 [3],[28],[41],[80], Hip1R [5], epsin [4],[6], actin [18], endophilin2 [37], synaptojanin1 [37], APPL1 [59],[60], OCRL1 [59], FBP17 [6], N-WASP [47], Arp3 [47],[80], GAK [15],[16], and dynamin1/2 [3],[15],[18],[41] to endocytic sites. This enabled CCP/CCV components to be divided into those that are recruited at early stages (orange time course in [A]) or transiently at late stages (yellow time course in [A]). In addition, some studies referenced the recruitment of pairs of endocytic proteins to one another. For example, the recruitment of SNX9 was found to occur over a similar time course to dynamin1 [19], while GAK was recruited to sites of endocytosis after dynamin1 [15]. Despite these efforts, the recruitment dynamics of many endocytic proteins remain poorly characterised and the precise timing of recruitment of endocytic proteins relative to scission remains unexplored.
(1.23 MB TIF)

Figure S2 **Detection and timing of single scission events using TIR-FM and the ppH assay.** (A) An adherent cell growing on the surface of a coverslip was imaged using TIR-FM [64]. A large-diameter perfusion tip (100 µm internal diameter, not to scale) was used to perfuse the target cell with buffer, alternating between buffer of pH 7.4 and pH 5.5 in successive images. (B) Example pairs of images acquired at arbitrary time points *t* and *t*+2 s of a cell expressing Clc-mCherry and TfR-phl. Both clathrin, tagged with mCherry (Clc-mCherry), and TfR, tagged with super-ecliptic phluorin (TfR-phl), fluoresced brightly at pH 7.4. Immediately following acquisition of the images at pH 7.4, the perfusate was switched to pH 5.5 buffer and a second set of images acquired. Clc-mCherry fluorescence was unaffected by the change in pH, while TfR-phl fluorescence at the plasma membrane quenched, revealing bright punctae of insulated (i.e., endocytosed) TfR-phl. (C) An example scission event from the cell shown in (B) focused on a short time window immediately before and after scission. A coated pit (Clc spot, lower panel) colocalized with a patch of TfR-phl (TfR7, top panel). From *t* = −12 s through *t* = −4 s the patch of TfR7 was accessible to extracellular acidification. Thereafter, the patch of TfR-phl became insulated from external acidification, indicating scission occurred in the preceding pulse of pH 7.4 buffer. The frame in which the acid-resistant TfR-phl spot appeared was subsequently defined as *t* = 0 s. Following scission, the newly formed CCV uncoated, indicated by loss of clathrin signal between *t* = 4 s and *t* = 12 s. Between *t* = 8 s and *t* = 12 s the insulated TfR-phl spot disappeared, indicating that the CCV either acidified or moved away from the plasma membrane. (D) Topological interpretation of key frames before and after scission indicated by blue box in (C). Fawn indicates cytosol; blue, extracellular buffer; red, clathrin; and green or black lollipops, TfR-phl. At *t* = −4 s the CCP was deeply invaginated, and TfR-phl was accessible to extracellular acidification (black lollipops). In the following image at pH 7.4 TfR-phl concentrated at the CCP and on the membrane fluoresced brightly (green lollipops). Between *t* = −4 s and *t* = −2 s scission occurred, insulating the receptor cargo from subsequent external acidification at *t* = 0 s. (E) Logic plot of image acquisition (upper trace, high indicates camera exposing), valve command (middle trace), and resulting change in pH at the cell. Note that detected scission events actually occurred between ∼−3.8 s and ∼−1.8 s (indicated in grey).
(2.31 MB TIF)

Figure S3 **Correlations between Clc, TfR7, and TfR5.** (A–C) Average normalised fluorescence traces for (A) TfR7, (B) TfR5, and (C) Clc for 851 scission events. Fluorescence traces were normalised by cell to control for cell-to-cell variability due to variable expression levels and/or differences in illumination. Grey bars indicate time intervals over which average fluorescence measurements were calculated for individual normalised fluorescence traces, and which were subsequently used as test statistics to measure correlations. For TfR5 the peak fluorescence at scission was corrected for incomplete quenching (i.e., *F*_TfR5_ = *F*_2_−*F*_1_). (D and E) Scatter plots of (D) *F*_TfR7_ versus *F*_Clc_ (Spearman's rho = 0.85, *p*<0.05) and (E) *F*_TfR7_ versus *F*_TfR5_ (Spearman's rho = −0.0022, *p*>0.05). There was also no correlation between Clc and TfR5 fluorescence (not shown, Spearman's rho = −0.0024, *p*>0.05). Solid dark blue lines indicate locally weighted smoothed regressions to visualise overall trends (smoothing factor = 0.75). Dotted dark blue lines indicate 95% confidence intervals. From this we conclude that larger CCSs harboured more TfR-phl cargo and that TfR-phl can be used as a surrogate signal to indicate CCS size. However, there was no correlation between CCS size and the amount of TfR-phl internalized by scission events. (F–I) Thin section EM images of clathrin-coated invaginations in NIH-3T3 cells, (F) without TfR-phl expression, (G) with TfR-phl expression at pH 7.4, and (H and I) with TfR-phl expression after incubation at pH 5.5 for (H) 1 min and (I) 10 min.
(4.06 MB TIF)

Figure S4 **Correction for bleed through, the calculation of confidence intervals, and the recruitment signature of Cav1-mRFP as a negative control.** (A) Averages of fluorescent measures for Dyn1-mCherry for one cell (290 events) at pH 5.5 (red) and pH 7.4 (brown). Note the offset between the two measures, most prominent before scission, when the TfR7 signal was greatest. (B) Average of the same events after bleed-through correction. For this cell, the bleed-through value (defined as the minimum of the sum of squared difference between the curves at the two pHs, inset) was 3.2%. (C) Example of shifts used to generate the randomized dataset. White area represents the central part of a cell, and the black area, the cell edges, as determined by a maximum projection of the TfR7 movie over time. Blue dots correspond to event coordinates. Starting from each blue dot, the red segments indicate the shift in the coordinates used to generate the randomized data. (D) Average fluorescent measures for the shifted events shown in (C) (green, TfR7; light green, TfR5; red, Dyn1-mCherry). Black lines show the median and 95% confidence intervals for 200 shifts. (E) A scission event in cells expressing Cav1-mCherry (note the Cav1 punctate structure). (F) Fluorescent measurements for TfR7, TfR5, and Cav1 corresponding to the event shown in (E). The circled points on the fluorescence traces correspond to the images shown in (E). Vertical blue line shows time = 0 and horizontal lines show fluorescence = 0. The horizontal scale bar is equivalent to 20 s. Note the negative fluorescent values for the Cav1-mCherry signal associated with the scission event in (E). (G) Average Cav1, TfR7, and TfR5 signal from all positive scission events (five cells, 846 events). The Cav1 signal associated with scission (red line, bottom panel) is below the median fluorescence values associated with random events (middle black line, bottom panel) and negative for the time points preceding scission, indicating Cav1-mCherry was excluded from areas of the plasma membrane associated with scission. (H) A schematic illustration of how a negative fluorescent recruitment signature arises. When a RFP-tagged protein is physically excluded by a CCS from the region of interest, centred upon the scission event (green spot in centre of region of interest), but is present outside of the region of interest (shown as a red spots), the average red channel fluorescence of the annulus (Fl_Ann_) will be greater than the average fluorescence value for the region of interest (Fl_ROI_), resulting in a “negative fluorescent” recruitment signature (i.e., Fl_ROI_−Fl_Ann_<0).
(1.38 MB TIF)

Figure S5 **Domain plots of the endocytic proteins analysed in this study.** The identity, start point, and end point of domains were obtained from http://www.pfam.org.
(1.71 MB TIF)

Figure S6 **Expression analysis of the proteins assayed.** Capillary RT-PCR analysis revealed that 30 of the 34 proteins analysed using the ppH assay are expressed in NIH-3T3 fibroblasts. The exceptions are amphiphysin1 and FCHo1, which are only expressed in mouse brain (*), and ACK1 and CIP4, which are not expressed in either NIH-3T3 fibroblasts or mouse brain (**).
(2.22 MB TIF)

Figure S7 **Uptake of Tfn-A647 in transfected NIH-3T3 cells.** A subset of RFP fusion constructs was assayed to determine whether expression had any gross defect upon Tfn uptake. Transfected NIH-3T3 cells were imaged by confocal microscopy before (*t* = 0 min) and after (*t* = 30 min) incubation with human transferrin conjugated to Alexa-647 (Tfn-A647). Green, TfR-phl; magenta, Tfn-A647; red, RFP fusion. (A) TfR-phl alone. (B) TfR-phl and OCRL1. (C) TfR-phl and BIN1. (D) TfR-phl and SNX9. (E) TfR-phl and CIP4. (F) TfR-phl and myosin1E. (G) TfR-phl and CALM. Cells expressing the RFP fusion constructs were still able to internalize Tfn-A647.
(3.90 MB TIF)

Figure S8 **Terminal versus non-terminal average fluorescence traces.** Average recruitment signatures were generated for each of the mCherry fusion constructs, and each set of traces was divided into terminal and non-terminal traces (see Materials and Methods). For transiently recruited proteins (e.g., Synd2, GAK, and OCRL1), there was little difference in the fluorescence traces for terminal versus non-terminal events. For proteins that bind to clathrin directly (e.g., mu2), there was a difference between the average fluorescence recruitment signature for terminal and non-terminal events. For transiently recruited proteins, the minimal difference in kinetics between terminal and non-terminal events shows that the core machinery of invagination and scission was constant, irrespective of the behaviour of the associated clathrin patch.
(4.79 MB TIF)

Figure S9 **Peak histograms for recruitment signatures.** For each endocytic protein, the peak fluorescence of recruitment (defined as the biggest peak greater than six standard deviations of the last six *F*_RFP_ values of the recording) was plotted as a histogram.
(4.44 MB TIF)

Figure S10 **Comparison of recruitment signatures for automated analysis and “recalled” events.** To check that the automated analysis did not bias the selection of bona fide scission events, the sets of rejected events for examples cells expressing (A) Clc, (B) Hip1R, (C) N-WASP, (D) Dyn1, and (E) GAK were manually checked. Events deemed by a human observer to be bona fide were “recalled”. The recruitment signatures for (Ai–Ei) automatically selected events and (Aii–Eii) recalled events were indistinguishable. Therefore, the automated selection was not biased.
(2.24 MB TIF)

Table S1 **Parameters for the cells used in this study.**
(0.10 MB DOC)

Table S2 **Construct details.** To obtain the constructs used in this study please contact Addgene (http://www.addgene.org).
(0.08 MB DOC)

Video S1 **Example region of interest of cell shown in** **Figure 1A, 1C, and 1D** **.** Shows Clc-mCherry- (left) and TfR5-signal-reporting scission events (right).
(2.41 MB AVI)

## Abbreviations

CCP: clathrin-coated pit
CCS: clathrin-coated structure
CCV: clathrin-coated vesicle
CME: clathrin-mediated endocytosis
EM: electron microscopy
FP: fluorescent protein
GFP: green fluorescent protein
PI: phosphoinositide
ppH: pulsed pH
RFP: red fluorescent protein
RT-PCR: reverse transcription PCR
SNR: signal-to-noise ratio
TIR-FM: total internal reflection fluorescence microscopy
