# Supplementary figures and images for: A High Precision Survey of the Molecular Dynamics of Mammalian Clathrin-Mediated Endocytosis
Source: PLoS Biol. 2011 Mar 22;9(3):e1000604. doi: 10.1371/journal.pbio.1000604 (PMC3062526; doi:10.1371/journal.pbio.1000604)

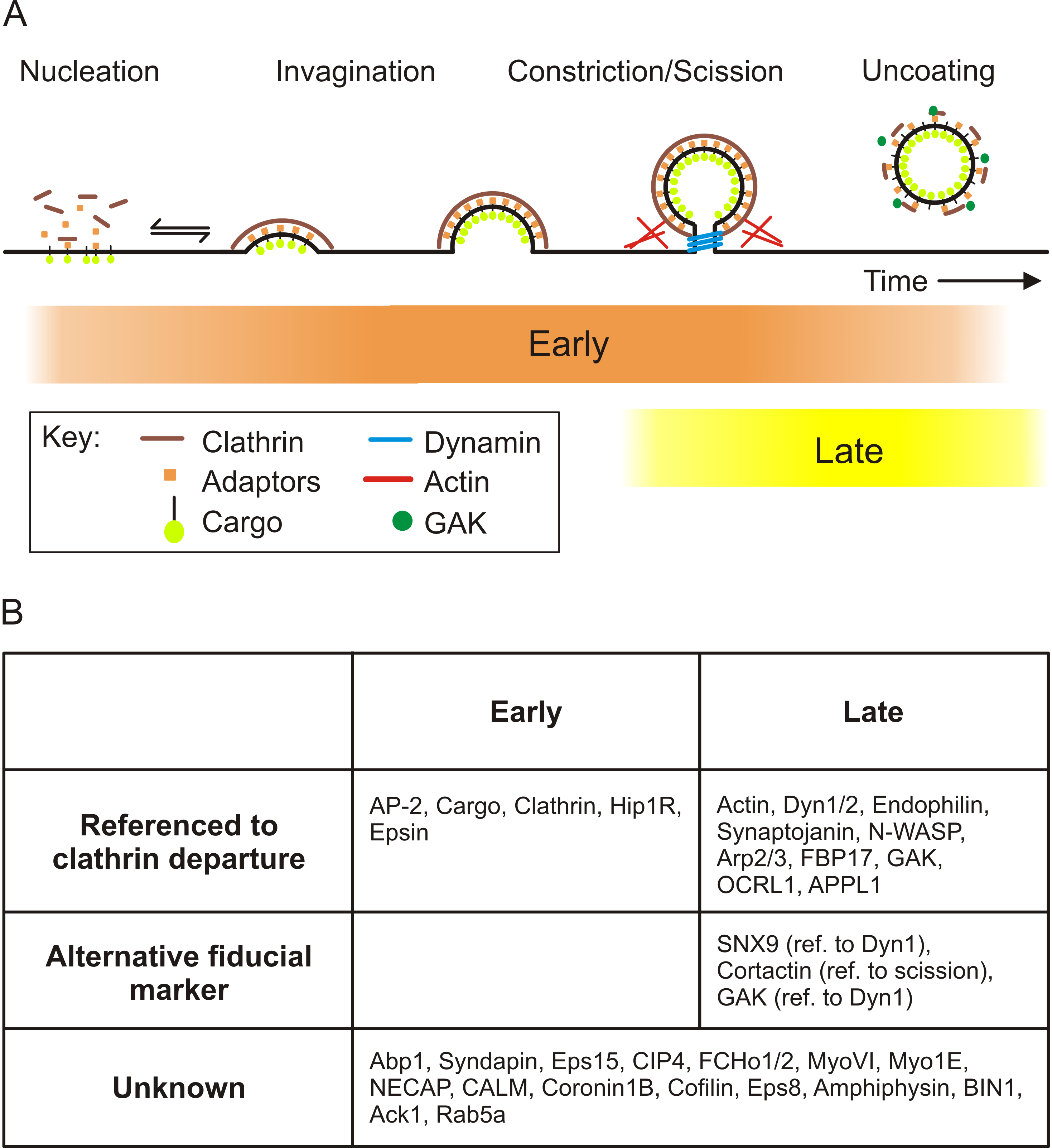

Supplement: Figure S1 — The canonical model of CME. (A) The formation of a CCV—as deduced from EM, genetic, biochemical, and live-cell fluorescent imaging data—begins with the random nucleation of a small patch of adaptors, the F-BAR domain protein FCHo, and clathrin at the plasma membrane that is stabilised (possibly by the acquisition of cargo [3],[28]) to form a CCP. The CCP invaginates and grows by the addition of adaptors and clathrin triskelion before the large GTPase dynamin constricts and pinches off the membrane neck, releasing a CCV. Following scission, the coated vesicle is stripped of the clathrin coat by the ATPase Hsc70 and cofactors and is subsequently processed by the endosomal machinery. (B) A table summarising the findings of live-cell imaging studies of endocytic protein dynamics in mammalian cells. In dual colour imaging experiments, different reference signals have been used to align and decipher the recruitment dynamics of fluorescently tagged endocytic proteins to sites of endocytosis. In a typical experiment the disappearance or increase in mobility of CCPs labelled with GFP or RFP clathrin light chain was used as an indicator for endocytosis. This approach was used to characterise the recruitment dynamics of AP2 [3],[28],[41],[80], Hip1R [5], epsin [4],[6], actin [18], endophilin2 [37], synaptojanin1 [37], APPL1 [59],[60], OCRL1 [59], FBP17 [6], N-WASP [47], Arp3 [47],[80], GAK [15],[16], and dynamin1/2 [3],[15],[18],[41] to endocytic sites. This enabled CCP/CCV components to be divided into those that are recruited at early stages (orange time course in [A]) or transiently at late stages (yellow time course in [A]). In addition, some studies referenced the recruitment of pairs of endocytic proteins to one another. For example, the recruitment of SNX9 was found to occur over a similar time course to dynamin1 [19], while GAK was recruited to sites of endocytosis after dynamin1 [15]. Despite these efforts, the recruitment dynamics of many endocytic proteins remain po [file pbio.1000604.s001.tif]

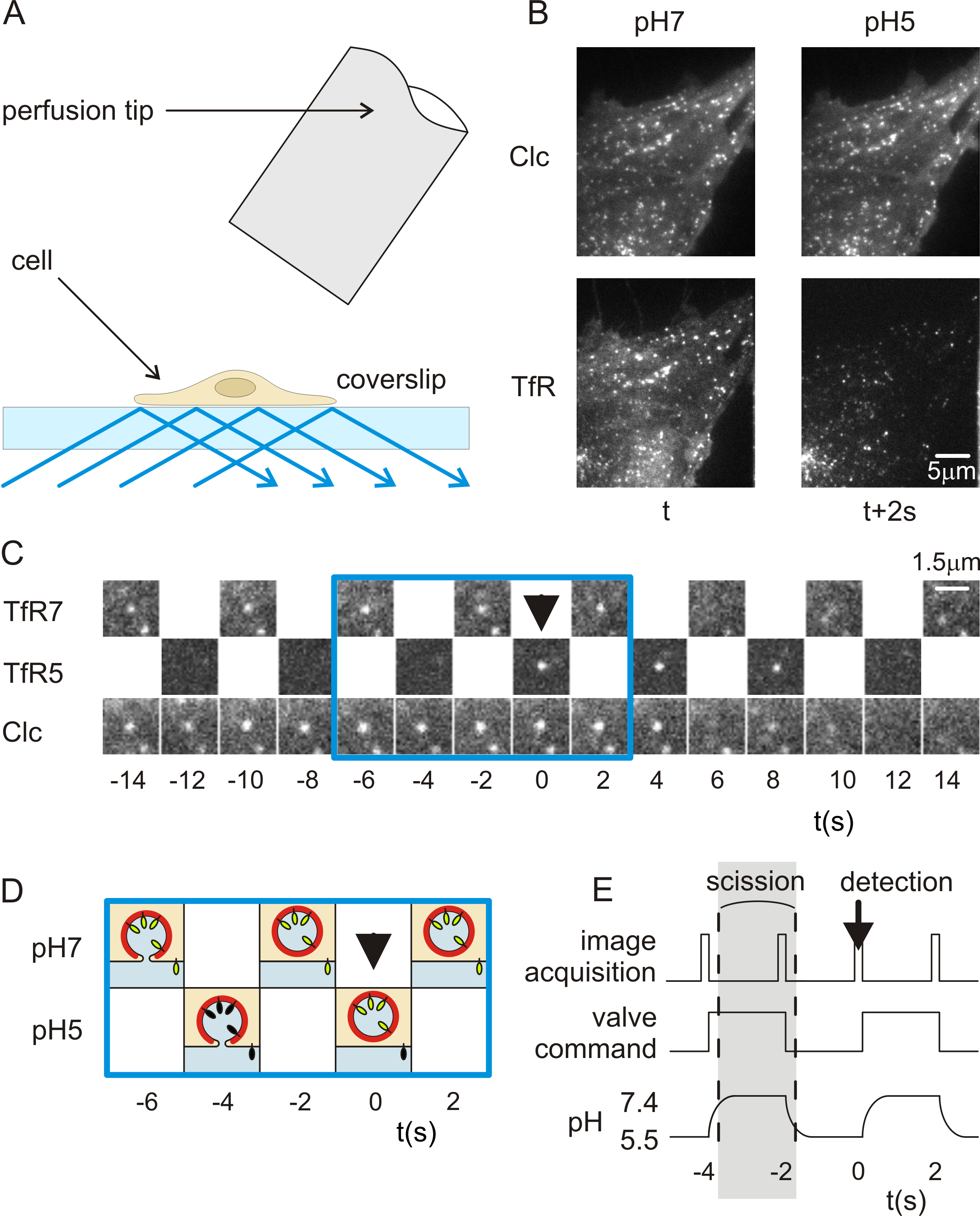

Supplement: Figure S2 — Detection and timing of single scission events using TIR-FM and the ppH assay. (A) An adherent cell growing on the surface of a coverslip was imaged using TIR-FM [64]. A large-diameter perfusion tip (100 µm internal diameter, not to scale) was used to perfuse the target cell with buffer, alternating between buffer of pH 7.4 and pH 5.5 in successive images. (B) Example pairs of images acquired at arbitrary time points t and t+2 s of a cell expressing Clc-mCherry and TfR-phl. Both clathrin, tagged with mCherry (Clc-mCherry), and TfR, tagged with super-ecliptic phluorin (TfR-phl), fluoresced brightly at pH 7.4. Immediately following acquisition of the images at pH 7.4, the perfusate was switched to pH 5.5 buffer and a second set of images acquired. Clc-mCherry fluorescence was unaffected by the change in pH, while TfR-phl fluorescence at the plasma membrane quenched, revealing bright punctae of insulated (i.e., endocytosed) TfR-phl. (C) An example scission event from the cell shown in (B) focused on a short time window immediately before and after scission. A coated pit (Clc spot, lower panel) colocalized with a patch of TfR-phl (TfR7, top panel). From t = −12 s through t = −4 s the patch of TfR7 was accessible to extracellular acidification. Thereafter, the patch of TfR-phl became insulated from external acidification, indicating scission occurred in the preceding pulse of pH 7.4 buffer. The frame in which the acid-resistant TfR-phl spot appeared was subsequently defined as t = 0 s. Following scission, the newly formed CCV uncoated, indicated by loss of clathrin signal between t = 4 s and t = 12 s. Between t = 8 s and t = 12 s the insulated TfR-phl spot disappeared, indicating that the CCV either acidified or moved away from the plasma membrane. (D) Topological interpretation of key frames before and after scission indicated by blue box in (C). Fawn indicates cytosol; blue, extracellular buffer; red, clathrin; and green or black lollipops, TfR-phl. At t = −4 s the CCP [file pbio.1000604.s002.tif]

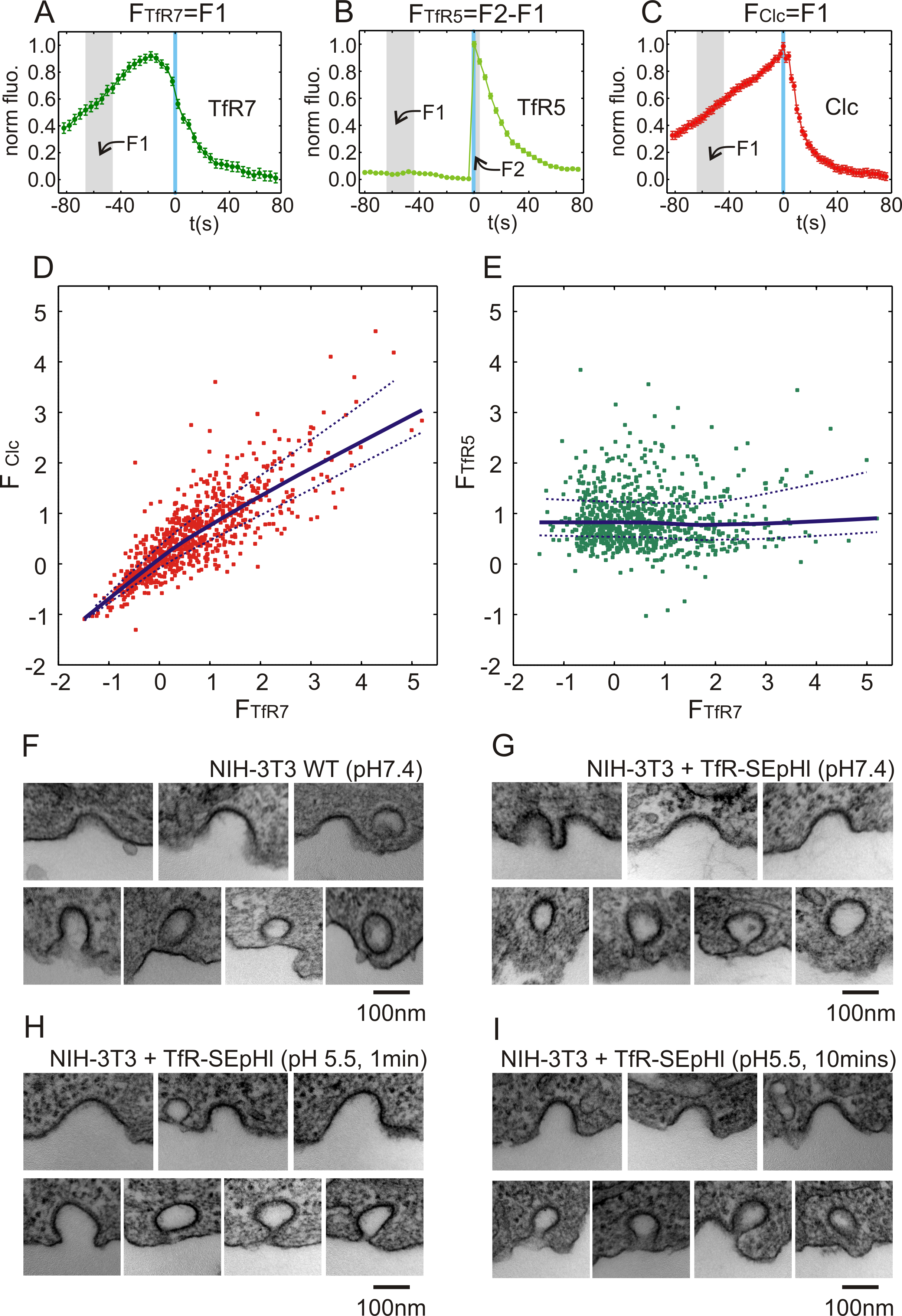

Supplement: Figure S3 — Correlations between Clc, TfR7, and TfR5. (A–C) Average normalised fluorescence traces for (A) TfR7, (B) TfR5, and (C) Clc for 851 scission events. Fluorescence traces were normalised by cell to control for cell-to-cell variability due to variable expression levels and/or differences in illumination. Grey bars indicate time intervals over which average fluorescence measurements were calculated for individual normalised fluorescence traces, and which were subsequently used as test statistics to measure correlations. For TfR5 the peak fluorescence at scission was corrected for incomplete quenching (i.e., F TfR5 = F 2−F 1). (D and E) Scatter plots of (D) F TfR7 versus F Clc (Spearman's rho = 0.85, p<0.05) and (E) F TfR7 versus F TfR5 (Spearman's rho = −0.0022, p>0.05). There was also no correlation between Clc and TfR5 fluorescence (not shown, Spearman's rho = −0.0024, p>0.05). Solid dark blue lines indicate locally weighted smoothed regressions to visualise overall trends (smoothing factor = 0.75). Dotted dark blue lines indicate 95% confidence intervals. From this we conclude that larger CCSs harboured more TfR-phl cargo and that TfR-phl can be used as a surrogate signal to indicate CCS size. However, there was no correlation between CCS size and the amount of TfR-phl internalized by scission events. (F–I) Thin section EM images of clathrin-coated invaginations in NIH-3T3 cells, (F) without TfR-phl expression, (G) with TfR-phl expression at pH 7.4, and (H and I) with TfR-phl expression after incubation at pH 5.5 for (H) 1 min and (I) 10 min. (4.06 MB TIF) [file pbio.1000604.s003.tif]

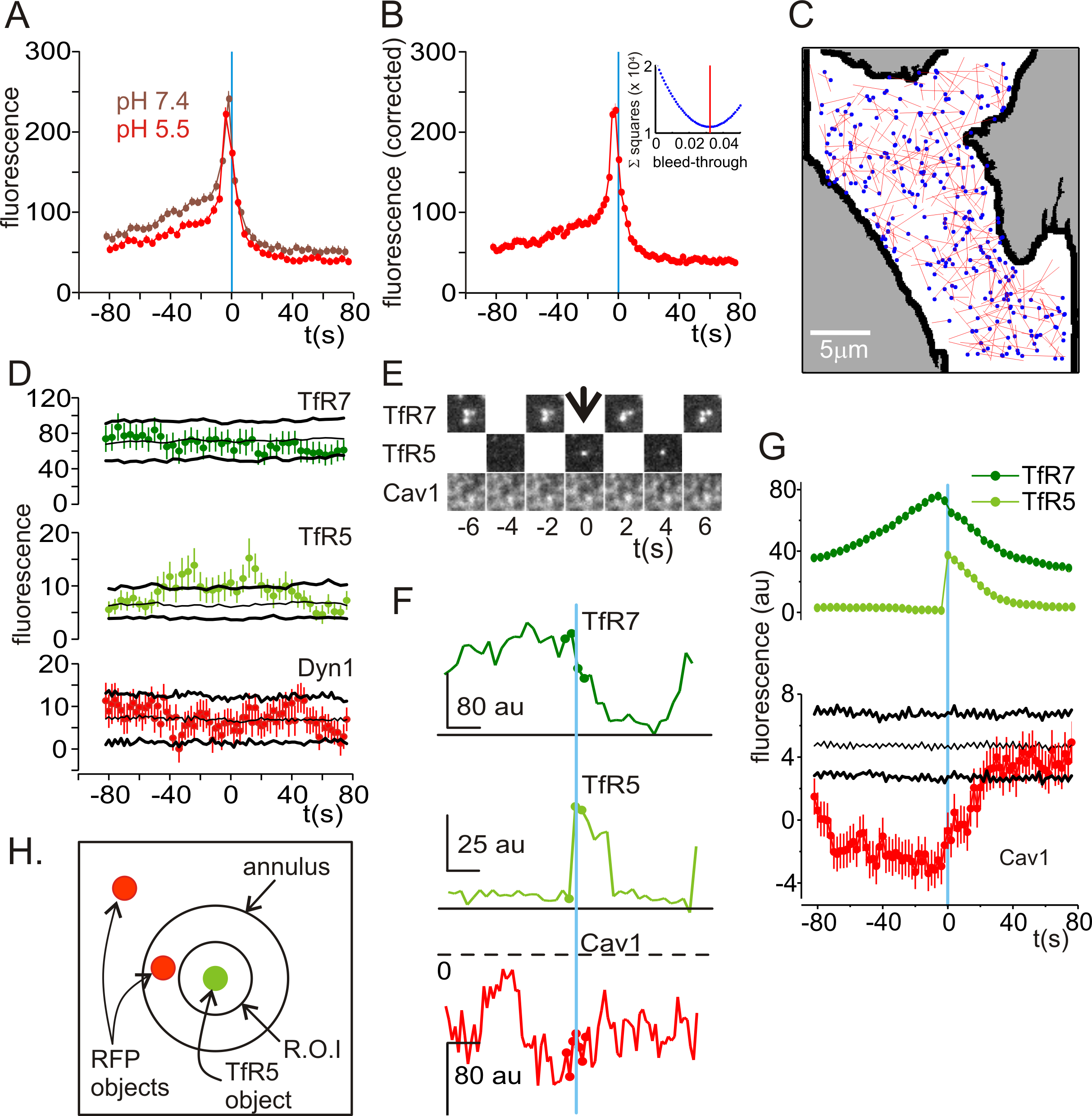

Supplement: Figure S4 — Correction for bleed through, the calculation of confidence intervals, and the recruitment signature of Cav1-mRFP as a negative control. (A) Averages of fluorescent measures for Dyn1-mCherry for one cell (290 events) at pH 5.5 (red) and pH 7.4 (brown). Note the offset between the two measures, most prominent before scission, when the TfR7 signal was greatest. (B) Average of the same events after bleed-through correction. For this cell, the bleed-through value (defined as the minimum of the sum of squared difference between the curves at the two pHs, inset) was 3.2%. (C) Example of shifts used to generate the randomized dataset. White area represents the central part of a cell, and the black area, the cell edges, as determined by a maximum projection of the TfR7 movie over time. Blue dots correspond to event coordinates. Starting from each blue dot, the red segments indicate the shift in the coordinates used to generate the randomized data. (D) Average fluorescent measures for the shifted events shown in (C) (green, TfR7; light green, TfR5; red, Dyn1-mCherry). Black lines show the median and 95% confidence intervals for 200 shifts. (E) A scission event in cells expressing Cav1-mCherry (note the Cav1 punctate structure). (F) Fluorescent measurements for TfR7, TfR5, and Cav1 corresponding to the event shown in (E). The circled points on the fluorescence traces correspond to the images shown in (E). Vertical blue line shows time = 0 and horizontal lines show fluorescence = 0. The horizontal scale bar is equivalent to 20 s. Note the negative fluorescent values for the Cav1-mCherry signal associated with the scission event in (E). (G) Average Cav1, TfR7, and TfR5 signal from all positive scission events (five cells, 846 events). The Cav1 signal associated with scission (red line, bottom panel) is below the median fluorescence values associated with random events (middle black line, bottom panel) and negative for the time points preceding scission, indicating Cav1-mCherry [file pbio.1000604.s004.tif]

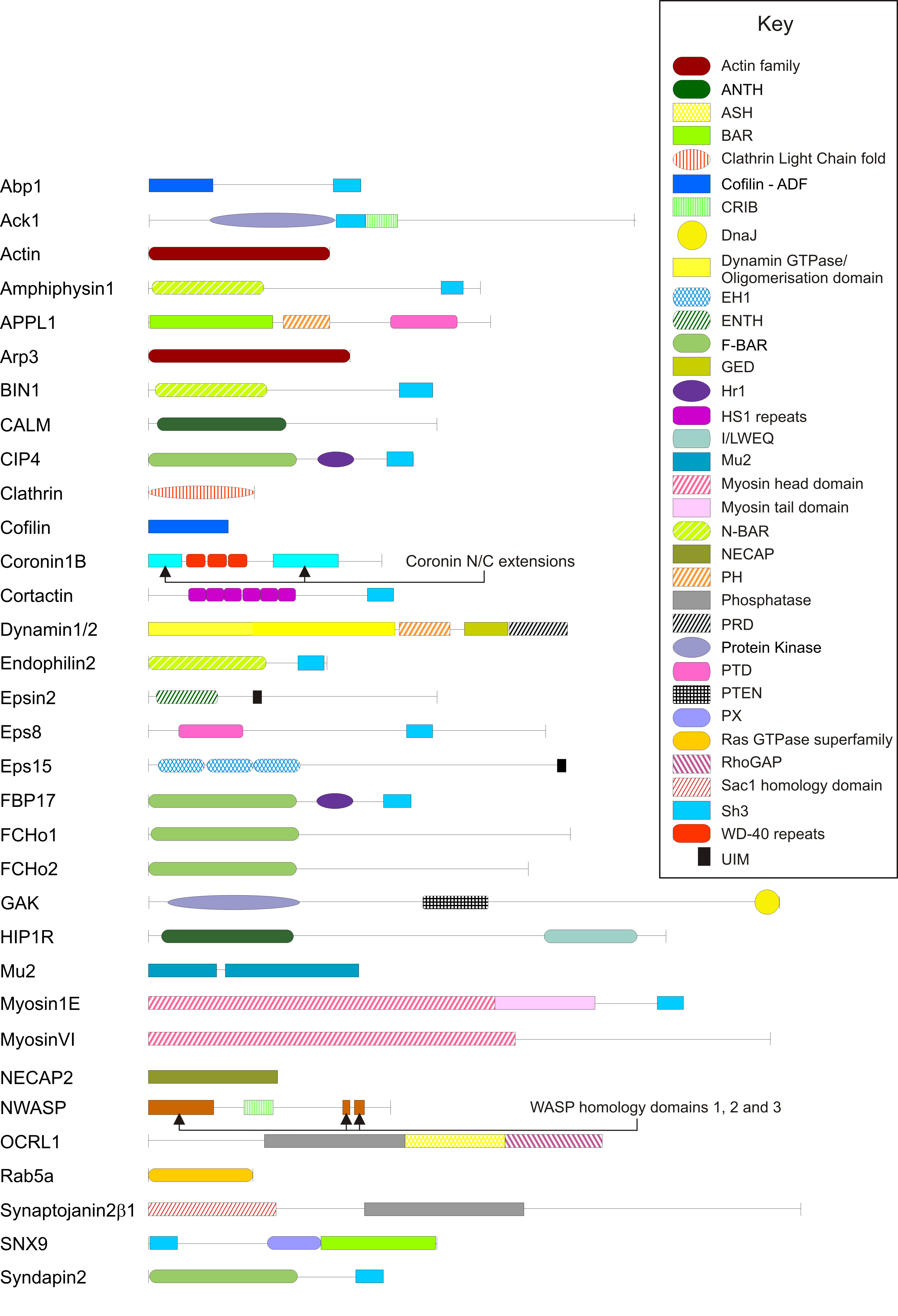

Supplement: Figure S5 — Domain plots of the endocytic proteins analysed in this study. The identity, start point, and end point of domains were obtained from http://www.pfam.org. (1.71 MB TIF) [file pbio.1000604.s005.tif]

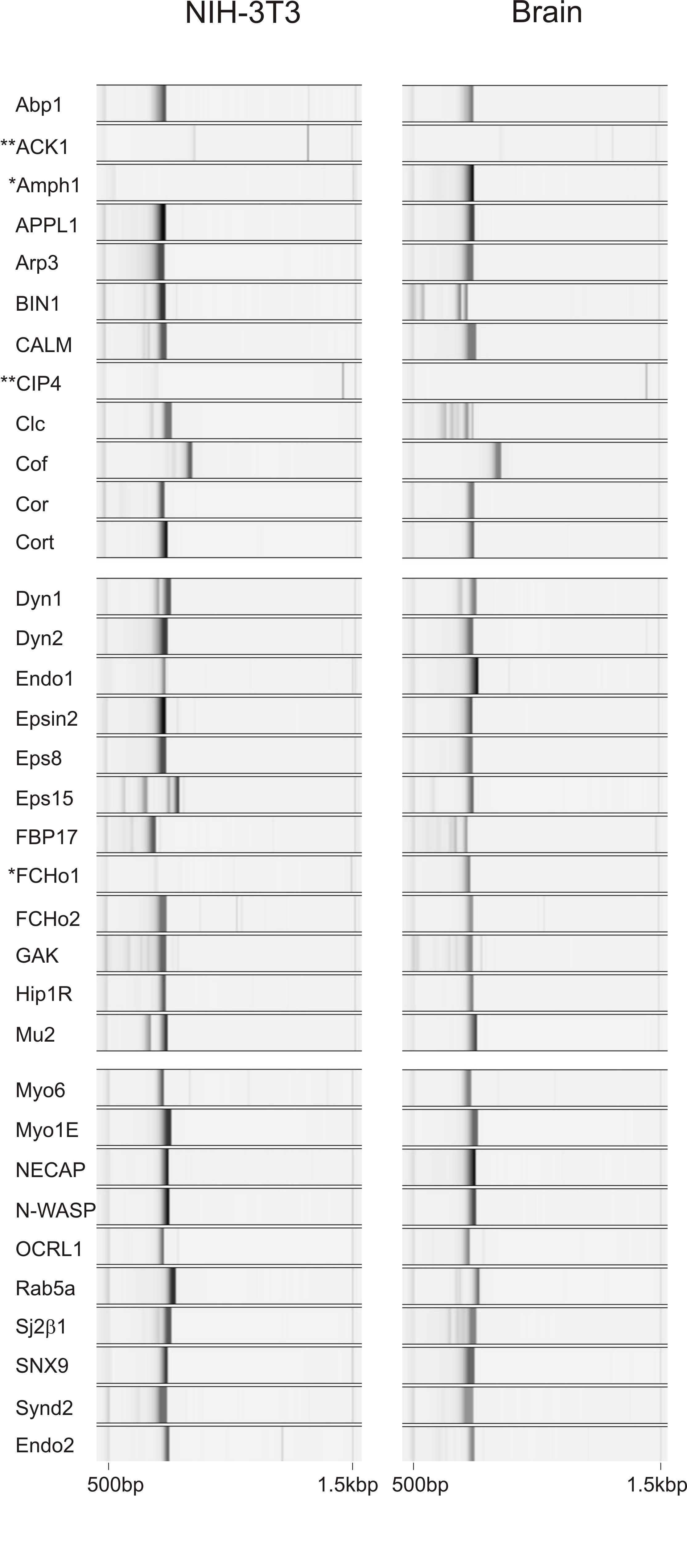

Supplement: Figure S6 — Expression analysis of the proteins assayed. Capillary RT-PCR analysis revealed that 30 of the 34 proteins analysed using the ppH assay are expressed in NIH-3T3 fibroblasts. The exceptions are amphiphysin1 and FCHo1, which are only expressed in mouse brain (*), and ACK1 and CIP4, which are not expressed in either NIH-3T3 fibroblasts or mouse brain (**). (2.22 MB TIF) [file pbio.1000604.s006.tif]

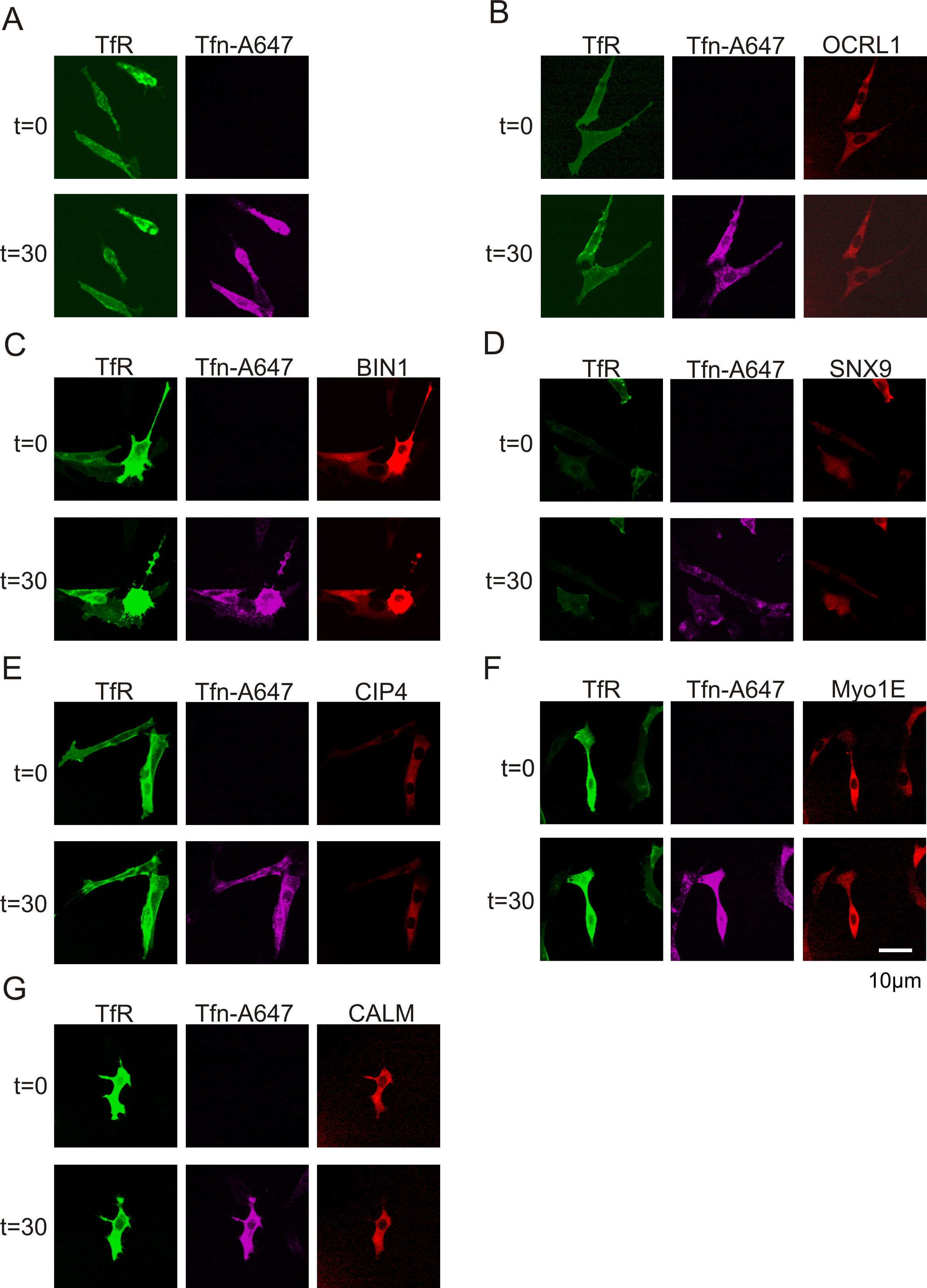

Supplement: Figure S7 — Uptake of Tfn-A647 in transfected NIH-3T3 cells. A subset of RFP fusion constructs was assayed to determine whether expression had any gross defect upon Tfn uptake. Transfected NIH-3T3 cells were imaged by confocal microscopy before (t = 0 min) and after (t = 30 min) incubation with human transferrin conjugated to Alexa-647 (Tfn-A647). Green, TfR-phl; magenta, Tfn-A647; red, RFP fusion. (A) TfR-phl alone. (B) TfR-phl and OCRL1. (C) TfR-phl and BIN1. (D) TfR-phl and SNX9. (E) TfR-phl and CIP4. (F) TfR-phl and myosin1E. (G) TfR-phl and CALM. Cells expressing the RFP fusion constructs were still able to internalize Tfn-A647. (3.90 MB TIF) [file pbio.1000604.s007.tif]

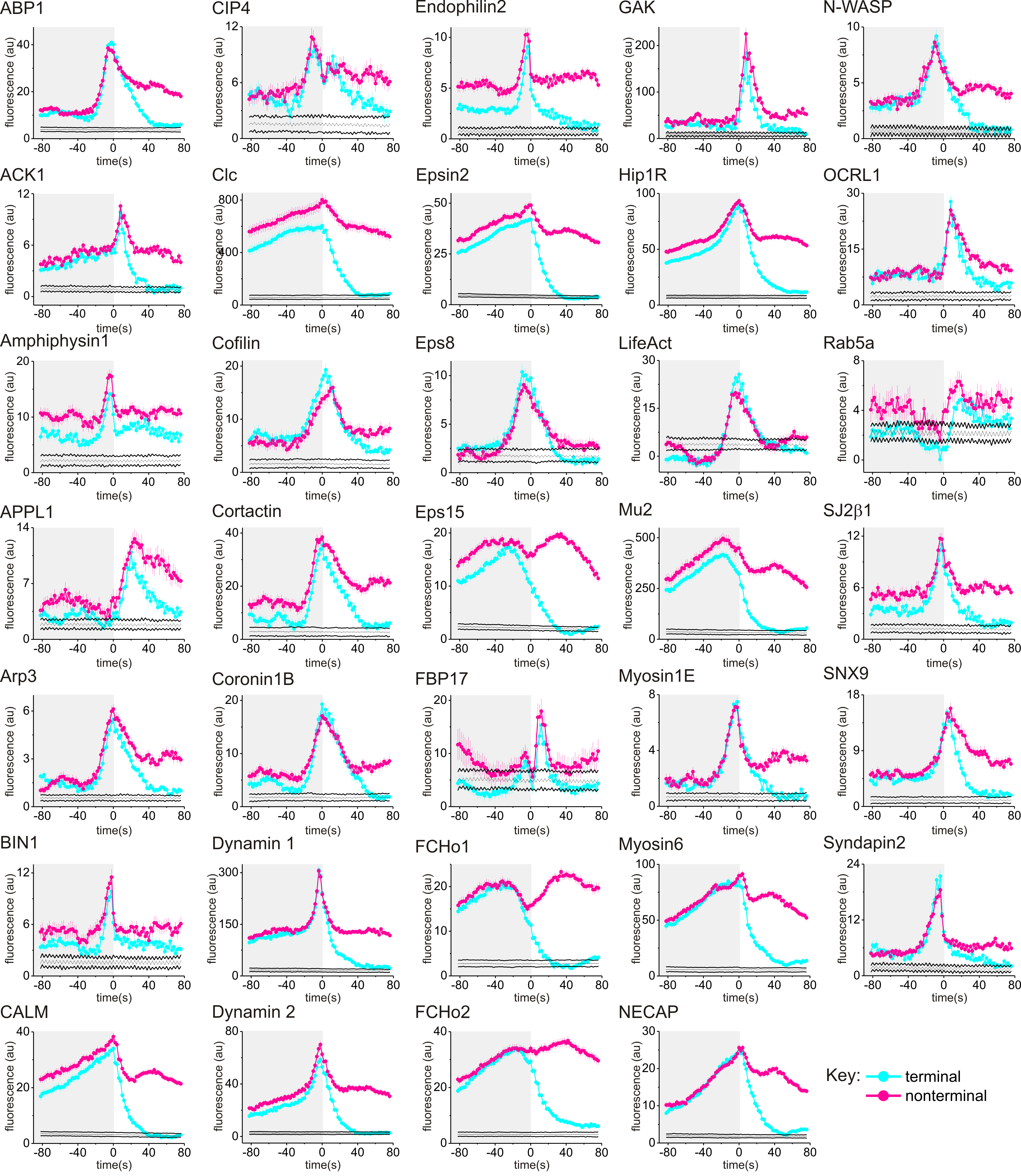

Supplement: Figure S8 — Terminal versus non-terminal average fluorescence traces. Average recruitment signatures were generated for each of the mCherry fusion constructs, and each set of traces was divided into terminal and non-terminal traces (see Materials and Methods). For transiently recruited proteins (e.g., Synd2, GAK, and OCRL1), there was little difference in the fluorescence traces for terminal versus non-terminal events. For proteins that bind to clathrin directly (e.g., mu2), there was a difference between the average fluorescence recruitment signature for terminal and non-terminal events. For transiently recruited proteins, the minimal difference in kinetics between terminal and non-terminal events shows that the core machinery of invagination and scission was constant, irrespective of the behaviour of the associated clathrin patch. (4.79 MB TIF) [file pbio.1000604.s008.tif]

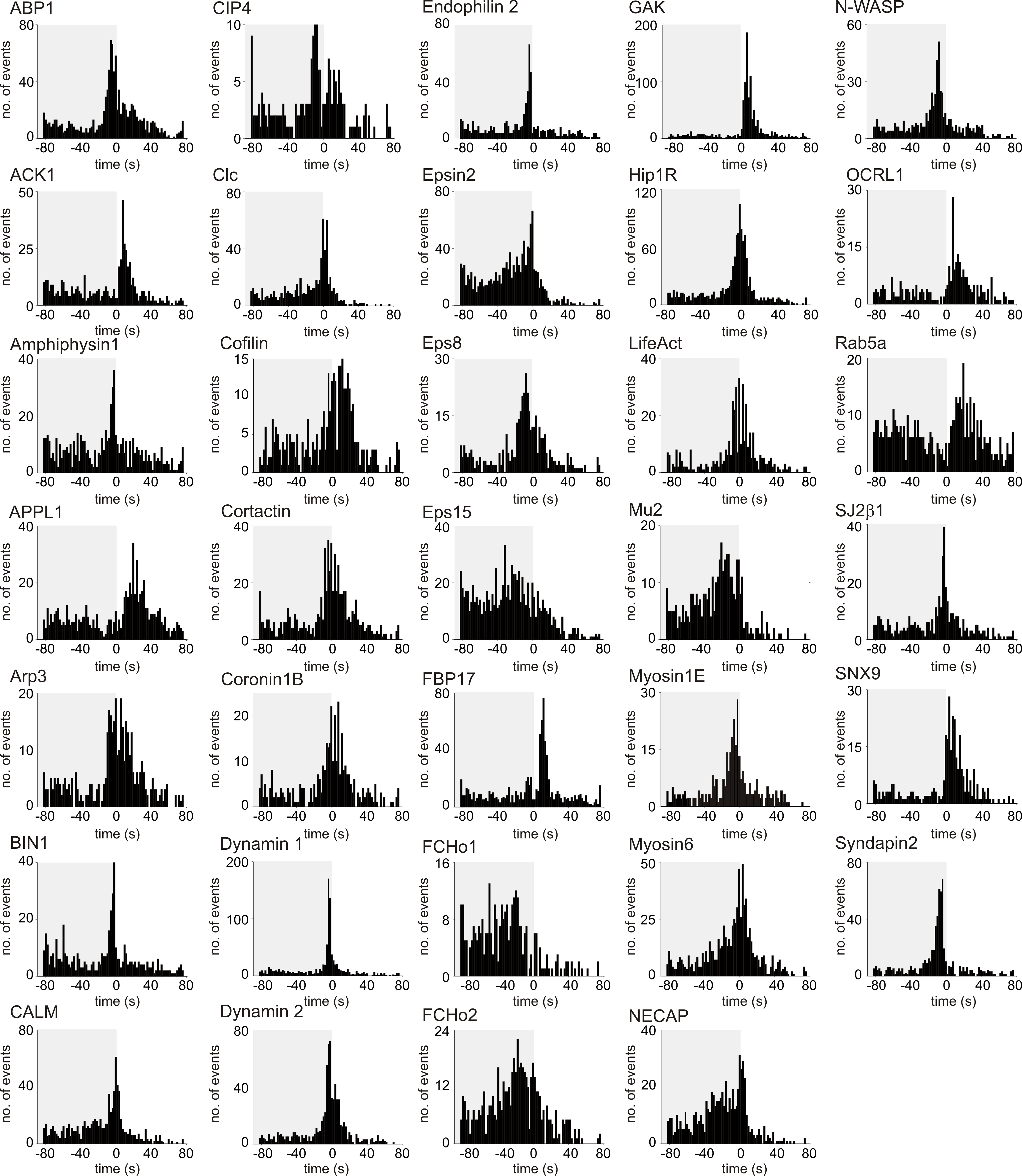

Supplement: Figure S9 — Peak histograms for recruitment signatures. For each endocytic protein, the peak fluorescence of recruitment (defined as the biggest peak greater than six standard deviations of the last six F RFP values of the recording) was plotted as a histogram. (4.44 MB TIF) [file pbio.1000604.s009.tif]

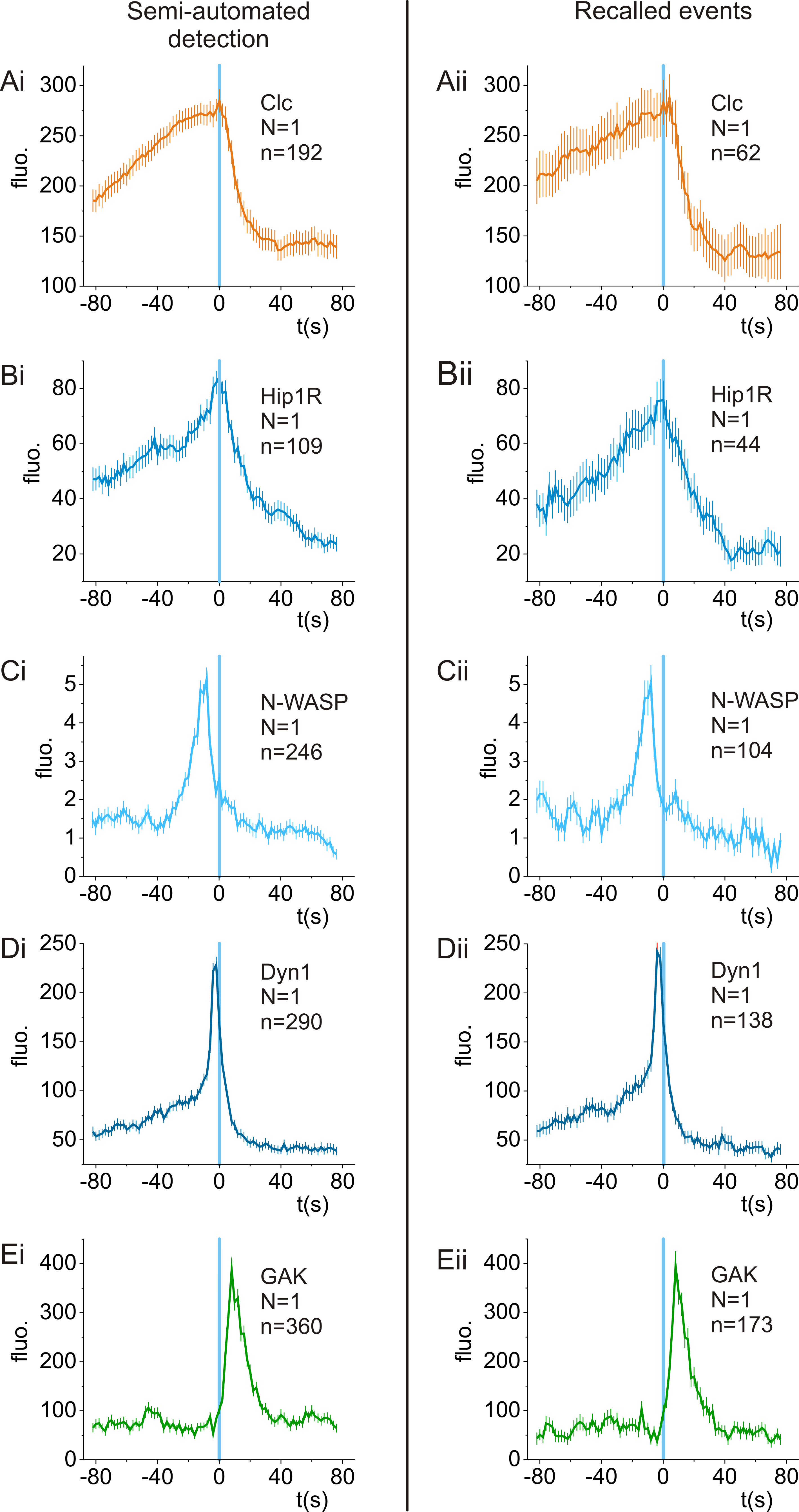

Supplement: Figure S10 — Comparison of recruitment signatures for automated analysis and “recalled” events. To check that the automated analysis did not bias the selection of bona fide scission events, the sets of rejected events for examples cells expressing (A) Clc, (B) Hip1R, (C) N-WASP, (D) Dyn1, and (E) GAK were manually checked. Events deemed by a human observer to be bona fide were “recalled”. The recruitment signatures for (Ai–Ei) automatically selected events and (Aii–Eii) recalled events were indistinguishable. Therefore, the automated selection was not biased. (2.24 MB TIF) [file pbio.1000604.s010.tif]
