# Supplementary material for: A High Precision Survey of the Molecular Dynamics of Mammalian Clathrin-Mediated Endocytosis
Source: PLoS Biol. 2011 Mar 22;9(3):e1000604. doi: 10.1371/journal.pbio.1000604 (PMC3062526; doi:10.1371/journal.pbio.1000604)
Supplement: Table S1 — Parameters for the cells used in this study. (0.10 MB DOC) [file pbio.1000604.s011.doc]

**Table S1. Parameters for the cells used in this study**

| Protein | #cells | #events | #Term | #Nterm | %Term | peak time | peak value | scission rate | | |
| --- | --- | --- | --- | --- | --- | --- | --- | --- | --- | --- |
|  |  |  |  |  |  | (s) | normalized | (event/100 µm²/min) | | |
| Abp1 | 5 | 1479 | 723 | 560 | 56 | -2 | 40.5 | 3.8 | ± | 1.6 |
| Ack1 | 5 | 935 | 397 | 408 | 49 | 8 | 30.8 | 2.6 | ± | 1.1 |
| amphiphysin1 | 5 | 1912 | 886 | 759 | 54 | -2 | 15.1 | 5.9 | ± | 1.3 |
| APPL1 | 5 | 1224 | 597 | 474 | 56 | 20 | 14.8 | 3.7 | ± | 1.2 |
| Arp3 | 7 | 985 | 479 | 372 | 56 | 0 | 29.3 | 1.9 | ± | 1.4 |
| BIN1(amph2) | 5 | 1333 | 746 | 425 | 64 | -2 | 14.4 | 4.2 | ± | 0.9 |
| CALM | 6 | 1030 | 436 | 468 | 48 | 0 | 44.1 | 2.4 | ± | 0.8 |
| CIP4(Toca3) | 5 | 701 | 324 | 266 | 55 | -10 | 10.2 | 2.5 | ± | 0.8 |
| cofilin | 5 | 822 | 458 | 265 | 63 | 8 | 20.3 | 2.3 | ± | 0.9 |
| coronin | 6 | 573 | 251 | 249 | 50 | 0 | 24.5 | 2.4 | ± | 1.3 |
| cortactin | 5 | 994 | 453 | 407 | 53 | 0 | 21.6 | 1.9 | ± | 1.5 |
| dynamin1 | 7 | 1278 | 533 | 571 | 48 | -4 | 60.0 | 2.5 | ± | 0.9 |
| dynamin2 | 6 | 944 | 539 | 305 | 64 | -2 | 57.8 | 4.2 | ± | 2.0 |
| endophilin2 | 6 | 1696 | 820 | 663 | 55 | -4 | 25.8 | 2.9 | ± | 1.1 |
| Eps15 | 7 | 1307 | 595 | 573 | 51 | -32 | 33.9 | 5.8 | ± | 2.9 |
| Eps8 | 5 | 863 | 436 | 302 | 59 | -10 | 11.1 | 4.0 | ± | 3.0 |
| epsin | 5 | 1701 | 895 | 597 | 60 | 0 | 57.5 | 2.6 | ± | 1.1 |
| FBP17 | 6 | 1657 | 672 | 743 | 47 | 12 | 6.5 | 4.5 | ± | 2.1 |
| FCHO1 | 5 | 580 | 271 | 229 | 54 | -32 | 23.0 | 5.4 | ± | 3.3 |
| FCHO2 | 6 | 873 | 368 | 366 | 50 | -16 | 38.5 | 2.3 | ± | 1.1 |
| GAK | 6 | 1470 | 617 | 645 | 42 | 8 | 43.6 | 4.0 | ± | 3.0 |
| hip1R | 6 | 1522 | 662 | 609 | 52 | 0 | 65.0 | 4.6 | ± | 1.7 |
| CLCa | 7 | 851 | 410 | 328 | 56 | 0 | 38.5 | 3.2 | ± | 1.5 |
| lifeact (F-actin) | 5 | 711 | 334 | 298 | 53 | 0 | 10.7 | 1.8 | ± | 1.4 |
| Mu2 (AP2) | 5 | 484 | 258 | 156 | 62 | -18 | 34.7 | 2.4 | ± | 0.7 |
| myosin1e | 5 | 1030 | 497 | 378 | 57 | -2 | 25.9 | 3.4 | ± | 2.1 |
| myosin6 | 7 | 1102 | 513 | 442 | 54 | 0 | 50.7 | 2.4 | ± | 1.4 |
| NECAP | 5 | 881 | 381 | 388 | 50 | 0 | 41.3 | 2.8 | ± | 1.4 |
| NWASP | 6 | 1332 | 606 | 567 | 52 | -8 | 23.2 | 3.9 | ± | 1.1 |
| OCRL1 | 6 | 501 | 189 | 247 | 43 | 8 | 23.9 | 1.2 | ± | 0.6 |
| Rab5a | 5 | 1166 | 607 | 428 | 59 | 16 | 5.0 | 4.0 | ± | 3.2 |
| SNX9 | 5 | 568 | 280 | 227 | 55 | 4 | 27.1 | 3.3 | ± | 1.1 |
| synaptojanin2 | 5 | 1238 | 556 | 521 | 52 | -2 | 22.0 | 2.2 | ± | 0.9 |
| syndapin2 | 6 | 940 | 369 | 447 | 45 | -4 | 25.4 | 2.8 | ± | 1.1 |
|  |  |  |  |  |  |  |  |  |  |  |
|  | average | 1079 |  |  | 54 |  | 29.9 | 3.2 |  |  |
|  | std | 375 |  |  | 6 |  | 15.9 | 1.1 |  |  |

**#cells**: number of cells recorded; **#events**: total number of events detected and analysed; **#Term**: number of events classified as terminal, i.e. with disappearance of the CCS (see methods); **#Nterm**: number of events classified as non-terminal (no CCS disappearance); **%Term**: percentage of terminal events; **Peak time**: time of maximum average fluorescence relative to CCV detection, in seconds; **Peak value, normalized**: value of maximum fluorescence, normalized to randomized measures (see methods for calculation); **Scission rate**: average over individual cells of the rate of event detection per µm^2^ per minute
