# Supplementary material for: A High Precision Survey of the Molecular Dynamics of Mammalian Clathrin-Mediated Endocytosis
Source: PLoS Biol. 2011 Mar 22;9(3):e1000604. doi: 10.1371/journal.pbio.1000604 (PMC3062526; doi:10.1371/journal.pbio.1000604)
Supplement: Table S2 — Construct details. To obtain the constructs used in this study please contact Addgene (http://www.addgene.org). (0.08 MB DOC) [file pbio.1000604.s012.doc]

**Table S2. Construct details.**

| **ORF**: | **Expression Vector:** | **Primers**: | **Source**: |
| --- | --- | --- | --- |
| Abp1 | tDimer-RFP-N1 | *For-*  GGGGACAAGTTTGTACAAAAAAGCAGGCTTAACCATGGCGGTGAACCTGAGCCGGAACG  *Rev-*  GGGGACCACTTTGTACAAGAAAGCTGGGTCCTCTATGAGCTCCACGTAGTTGGCAGG | Mus musculus, IMAGE clone 4986658 |
| Ack1 | pmCherry-C1 | *For-*  CGCAGATCTGGATCTGGTTCAGGAATGCAGCCGGAGGAGG  *Rev-*  GCGCGAATTCTCAGCGCTTGTGATGAGC | Mus musculus, IMAGE clone 6400209 |
| hArp3 | pmCherry-C1 | *For-*  GGGGACAAGTTTGTACAAAAAAGCAGGCTTAACCATGCCGGCATACCACTCTTCTCTCATG  *Rev-*  GGGGACCACTTTGTACAAGAAAGCTGGGTCCTGCCCAGGCCCCGAAAGACTCTTGTTC | Mus musculus, IMAGE clone 5261640 |
| Amphiphysin 1 | pmCherry-N1 | *For-*  GCGCGCGAATTCACCATGGCCGACATCAAGACG  *Rev-*  CGCGCCCCGGGTACTACTTCCACTACCAGTTGATCCTGTACCATGTCCCTCCAGGCGCCGCGTGAA | Mus musculus, IMAGE clone 5686478 |
| AP2 (µ2 subunit) | pIRESneoIII – mCherry inserted at residue 236 in linker region between N and C terminus | *For-*  GCGCGCGCTAGCACCATGATCGGAGGCTTATTCATCTAT  *Rev-*  GCGCGCACCGGTCTAGCAGCGGGTTTCGTAAATGCC | Rattus Norvegicus, a kind gift from David Owen. |
| APPL1 | pmCherry-C1 | *For*-  GCGCGCCTCGAGCTCGGATGGGTTCGACCTCGGGTTCGACCAAGGGTCAGCTGATGCCGGGGATCGACAAGCTG  *Rev*-  GCGCGCCCCGGGTTATGCTTCCGACTCTCTTTT | Mus musculus,  IMAGE clone 6511401 |
| BIN1 | pmCherry-N1 | *For-*  GCGCGCGAATTCACCATGGCAGAGATGGGGAGCAAG  *Rev-*  GCGCGCGGATCCCGCAGCTGACCCTTGGTCGAACCCGAGGTCGAACCCATCCGCTGCACCCGCTCTGTAAAATT | Mus musculus,  IMAGE clone  5718309 |
| CALM | pmCherry-N1 | *For*-  GCGCGCCTCGAGACCATGTCTGGCCAGAGCCTGACG  *Rev*-  GCGCGCCCCGGGCCAGCTGACCCTTGGTCGAACCCGAGGTCGAACCCATCCGCATAAACTGTATCTGTGCTCC | Mus musculus,  IMAGE clone  2651109 |
| Cav1 | pmCherry N1 | *NA* | A kind gift from Ben Nichols |
| CIP4 (TOCA 2) | pmCherry-C1 | *For*-  GCGCGCAGATCTCGGATGGGTTCGACCTCGGGTTCGACCAAGGGTCAGCTGATGGATTGGGGTACCGAGTTG  *Rev-*  GCGCGCGAATTCTCAGTTGAGTGTGACTCGGAG | Mus musculus  IMAGE clone  3500118 |
| Clathrin light chain | pmCherry-C1 | *For*-GCGCGCGAATTCTATGGCCGAGTTGGATCCATTC  *Rev-*  GCGCGCGGTACCTCAGTGCACCAGGGGGGCCTG | Mus musculus  IMAGE clone |
| Cofilin | pmCherry-C1 | *For-*  GCGCGCAGATCTCGGATGGGTTCGACCTCGGGTTCGACCAAGGGTCAGCTGATGGCCTCTGGTGTGGCTGTC  *Rev-*  GCGCGCGAATTCTCACAAAGGCTTGCCCTCCAG | Mus musculus,  Brain cDNA library |
| Coronin1B | pmCherry-N1 | *For*-  GCGCGCAGATCTACCATGTCCTTCCGAAAAGTTGTG  *Rev*-  GCGCGCGGATCCCGACTACTTCCACTACCAGTTGATCCTGTACCATGTCCTGTATCCCCATTCTCCATGCG | Mus musculus,  Brain cDNA library |
| Cortactin | pmCherry-C1 | NA | Mus musculus,  A kind gift from Natalie Soulet |
| Dynamin 1 | pmCherry-N1 | NA | Homo sapien, a kind gift from Wolf Almers |
| Dynamin 2 | pmCherry-N1 | *For-*  GCG CGC AGA TCT ACC ATG GGC AAC CGC GGG ATG GA  *Rev-*  GCGCGCGAATTCCACTACTTCCACTACCAGTTGATCCTGTACCATGTCCGTCGAGCAGGGATGGCTCGG | Homo sapien, IMAGE clone 6473791 |
| Endophilin 2 | pmCherry-N1 | NA | Rattus novegicus, a kind gift from Pietro de Camilli |
| Epsin 2 | pmCherry-C1 | *For-*  GCGCGCTCTCGAGCTCGGATGGGTTCGACCTCGGGTTCGACCAAGGGTCAGCTGATGACAACTTCATCTATCAG  *Rev-*  GCGCGCGGATCCCTAGAGAAGGAAAGGGTTTGT | Mus musculus,  IMAGE clone  2647379 |
| Eps8 | pmCherry-C1 | *For-*  GCGCGCGAATTCTCGGATGGGTTCGACCTCGGGTTCGACCAAGGGTCAGCTGATGAATGGTCATATGTCTAAC  *Rev-*  GCGCGCCCCGGGTCAGTGGCTGCTCCCTTCATC | Mus musculus,  IMAGE clone  4240899 |
| Eps15 | pmCherry-N1 | *For*-  GCGCGCCTCGAGACCATGGCTGCGGCGGCCCAGCTCTCCCTG  *Rev*-  GCGCGCCCCGGGCCAGCTGACCCTTGGTCGAACCCGAGGTCGAACCCATCCGTGCTTCTGATATCTCAGATTT | Rattus norvegicus, gift from H. McMahon lab |
| FBP-17 (TOCA 3) | pmCherry-C1 | *For*-  GCGCGCAGATCTCGGATGGGTTCGACCTCGGGTTCGACCAAGGGTCAGCTGATGAGCTGGGGCACCGAGCTC  *Rev*-  GCGCGCGAATTCCTAGGAATCTTTGGCATTTTT | Homo sapien,  Human kidney cDNA |
| FCHo1 | pmCherry-C1 | *For*-  GCGCGCAGATCTCGGATGGGTTCGACCTCGGGTTCGACCAAGGGTCAGCTGATGTCCTATTTTGGGGAACAT  *Rev*-  GCGCGCGAATTCTCAGCAGCTGACAAGGTACAT | Mus musculus, IMAGE clone 5323527 |
| FCHo2 | pmCherry-C1 | *For*-  GCGCGTCTCGAGCTCGGATGGGTTCGACCTCGGGTTCGACCAAGGGTCAGCTGATGAAGCATGGACAGATATCA  *Rev*-  GCGCGCGTCGACTCAACAATCTGCCAAGTATCG | Mus musculus IMAGE clone 6830607 |
| GAK | pmCherry-C1 | *For-*  GCTCGAGAATTCACCATGTCGCTGCTGCAGTCTGC  *Rev-*  GCGCTACCGGTGTACTACTTCCACTACCAGTTGATCCTGTACCATGTCCGAAGAGGGGCCTCGAGC | Mus musculus IMAGE clone 5705695 |
| Grb2 | pmCherry-C1 | *For-*  GCGCGCGAATTCACCATGGCCGACATCAAGACG  *Rev-*  CGCGCCCCGGGTACTACTTCCACTACCAGTTGATCCTGTACCATGTCCCTCCAGGCGCCGCGTGAA | Mus musculus IMAGE clone 5343274 |
| Hip1R | ptDimer-N1 | *For*-  GGGGACAAGTTTGTACAAAAAAGCAGGCTTAACCATGAACAGCATCAAGAATGTGCCGGCG  *Rev*-  GGGGACCACTTTGTACAAGAAAGCTGGGTCGTAGTTCACAAGTTGAGCTGGGTAGAC | Mus musculus IMAGE clone 30535741 |
| LifeAct | pmCherry-N1 | *For-*  AGCTTACCATGGGAGTGGCGGACCTCATCAAGAAGTTCGAGAGTATCAGTAAGGAGGAGCTGCA  *Rev-*  GCTCCTCCTTACTGATACTCTCGAACTTCTTGATGAGGTCCGCCACTCCCATGGTA | See …. |
| Myo1E | pmApple-C1 | *For*-  GCGCGCCTCGAGCTGGACATGGTACAGGATCAACTGGTAGTGGAAGTAGTATGGGCAGCAAAGGCGCCTAC  *Rev*-  GCGCGCGTCGACTCAGATCTTGGTGACGTAGTT | Mus musculus IMAGE clone 5053090 |
| MyoVI | pmCherry-C1 | *For-*GCGCGCCTCGAGCTGGACATGGTACAGGATCAACTGGTAGTGGAAGTAGTATGGAGGATGGAAAGCCCGTT  *Rev-*  GCGCGCGAATTCCTACTTTAACAGACTCTGCAG | Mus musculus, a kind gift from the laboratory of Folma Buss |
| NECAP1 | pmCherry-C1 | *For*-  GCGCGCAGATCTCGGATGGGTTCGACCTCGGGTTCGACCAAGGGTCAGCTGATGGCGGCAGAGCTGGAATAT  *Rev*-  GCGCGCGAATTCTCAAAACTGGACCCAGTTAGA | Mus musculus IMAGE clone 4013043 |
| OCRL1 | pmCherry-C1 | *For*-  GCGCGCCTCGAGCTCGGATGGGTTCGACCTCGGGTTCGACCAAGGGTCAGCTGATGGAGCCGCGGCTCCCGATT  *Rev*-  GCGCGCCCCGGGTTAGTCTTCCTCGTTCCCAAGCAG | Mus musculus IMAGE clone 30533468 |
| Rab5a | pmCherry-C1 | *For-*  GCGCAGATCTGGTTCAGGATCTGGAATGGCTAATCGAGGAGCAACA  *Rev-*  CGCGCGAATTCTCAGTTACTACAACACTGGCTTCTG | Mus musculus,  IMAGE clone:2649317 |
| SNX-9 | pmCherry-C1 | *For*-  GCGCGAGATCTGGTTCTTCAGGATCATCCGGTATGGCCACCAAGGCTCGG  *Rev-*  CGCGCGTCGACTTACATAACCGGGAAGCGG | Mus musculus IMAGE clone: 3257422 |
| Synaptojanin2 | pmCherry-C1 | *For*-  GCGCGCGTCGACCGGATGGGTTCGACCTCGGGTTCGACCAAGGGTCAGCTGATGGCTCTAAGCAAAGGGCTG  *Rev*-  GCGCGCGGATCCTCAAACACCTAATGTTGTCCT | Mus musculs IMAGE clone: 6390688 |
| Syndapin II | pmCherry-C1 | *For-*  GCGCGGAATTCTTCTGGAGGTTCTGGTATGTCTGTCACCTACGATGACTCTG  *Rev-*  CGCGCGGATCCTCACTGGATAGCCTCGACATAGTT | PCR from cDNA derived from 3T3s. |

Construct details including: open reading frame (ORF) cloned, target expression vector, primers used a
